# Identification of small molecule modulators of HIV-1 Tat and Rev protein accumulation

**DOI:** 10.1186/s12977-017-0330-0

**Published:** 2017-01-26

**Authors:** Ahalya Balachandran, Raymond Wong, Peter Stoilov, Sandy Pan, Benjamin Blencowe, Peter Cheung, P. Richard Harrigan, Alan Cochrane

**Affiliations:** 1 Department of Molecular Genetics, University of Toronto, 1 King’s College Circle, Toronto, ON M5S1A8 Canada; 2 Department of Laboratory Medicine and Pathobiology, University of Toronto, Toronto, ON Canada; 3 Department of Biochemistry, University of West Virginia, Morgantown, WV USA; 4 Donnelly Centre, University of Toronto, Toronto, ON Canada; 5 British Columbia Centre for Excellence in HIV/AIDS, 608-1081 Burrard St., Vancouver, BC Canada; 6 Department of Medicine, University of British Columbia, Vancouver, BC Canada

**Keywords:** HIV-1, RNA processing, Tat, Rev, Small molecule inhibitors

## Abstract

**Background:**

HIV-1 replication is critically dependent upon controlled processing of its RNA and the activities provided by its encoded regulatory factors Tat and Rev. A screen of small molecule modulators of RNA processing identified several which inhibited virus gene expression, affecting both relative abundance of specific HIV-1 RNAs and the levels of Tat and Rev proteins.

**Results:**

The screen for small molecules modulators of HIV-1 gene expression at the post-transcriptional level identified three (a pyrimidin-7-amine, biphenylcarboxamide, and benzohydrazide, designated 791, 833, and 892, respectively) that not only reduce expression of HIV-1 Gag and Env and alter the accumulation of viral RNAs, but also dramatically decrease Tat and Rev levels. Analyses of viral RNA levels by qRTPCR and RTPCR indicated that the loss of either protein could not be attributed to changes in abundance of the mRNAs encoding these factors. However, addition of the proteasome inhibitor MG132 did result in significant restoration of Tat expression, indicating that the compounds are affecting Tat synthesis and/or degradation. Tests in the context of replicating HIV-1 in PBMCs confirmed that 791 significantly reduced virus replication. Parallel analyses of the effect of the compounds on host gene expression revealed only minor changes in either mRNA abundance or alternative splicing. Subsequent tests suggest that 791 may function by reducing levels of the Tat/Rev chaperone Nap1.

**Conclusions:**

The three compounds examined (791, 833, 892), despite their lack of structural similarity, all suppressed HIV-1 gene expression by preventing accumulation of two key HIV-1 regulatory factors, Tat and Rev. These findings demonstrate that selective disruption of HIV-1 gene expression can be achieved.

**Electronic supplementary material:**

The online version of this article (doi:10.1186/s12977-017-0330-0) contains supplementary material, which is available to authorized users.

## Background

HIV-1 is heavily dependent upon the host cell RNA processing machinery for production of new virions. Following integration, transcription of the HIV-1 provirus generates a single 9 kb transcript that is subsequently processed through alternative splicing into over 40 mRNAs that fall into three classes; (1) unspliced (US), 9 kb mRNA encoding Gag and Gagpol, (2) singly spliced (SS), 4 kb mRNAs used to synthesize Vif, Vpr, Vpu, and Env, and (3) multiply spliced (MS), 1.8 kb mRNAs used to generate Tat, Rev, and Nef [1]. Extent of splicing of the primary transcript is regulated by the efficiency of the signals that form the splice sites themselves as well as the presence of adjacent regulatory elements that act to either promote (exon splicing enhancers, ESEs) or suppress (intron splicing silencers, ISS, or exon splicing silencer, ESS) use of the adjacent splice site [1]. The observation that mutation of a subset of these regulatory elements (ESSV, ESE_tat_) leads to dramatic perturbation in the viral mRNAs generated and substantially reduces virus replication underlines the significance of these splicing control mechanisms [2–4]. These findings also raise the possibility that manipulation of HIV-1 RNA processing could be used as an antiviral strategy.

Multiple host factors contribute to the regulation of HIV-1 RNA processing, many of which belong to the SR and hnRNP family of splicing regulators [1]. Both overexpression and depletion studies have highlighted several members of each family that play pivotal roles in regulating HIV-1 gene expression and virus replication [2, 5–11]. More relevant to the goal of the development of therapeutics, several groups have identified small molecules (digoxin, chlorhexidine, IDC16, ABX464, 8-azaguanine, 5310150, 1C8) which appear to function at different stages of HIV-1 RNA processing/expression to block viral structural protein expression [12–17]. Both digoxin and chlorhexidine were found to induce significant alterations in HIV-1 RNA abundance, with reductions in accumulation of both viral US and SS RNAs with no change/increased accumulation of MS RNAs [12, 14]. Both compounds dramatically reduced Gag, Env, and Rev expression with limited effects on Tat. In contrast, 8-azaguanine and 5350150 had similar effects as digoxin and chlorhexidine on HIV-1 RNA accumulation and Gag/Env synthesis but did not alter Tat or Rev levels [13]. Rather, both compounds altered Rev subcellular distribution putatively resulting in impaired Rev function. In contrast to the compounds listed above, IDC16 functions by inhibiting generation of HIV-1 MS RNA without affecting US RNA levels, possibly by altering the function of SRSF1 [15]. Consequently, there appear to be multiple mechanisms/stages post-integration at which small molecules can act to impair expression of HIV-1 structural proteins required for the assembly of new virions.

To expand the catalog of compounds affecting HIV-1 RNA processing/gene expression, we have tested a library of RNA splicing modulators identified in a cell based assay using alternative splicing of SMN2 as a reporter (P. Stoilov, unpublished data). Of the compounds tested, we report here the characterization of three, 3-(4-chlorophenyl)-5-methyl-*N*-(3-pyridinylmethyl)pyrazolo[1,5-a]pyrimidin-7-amine (designated 9147791 or 791), *N*-([2-(2-hydroxybenzoyl)hydrazino]carbonothioyl}-4-biphenylcarboxamide (designated 5227833 or 833), and 2-hydroxy-*N*′-(3-hydroxy-4-methoxybenzylidene)benzohydrazide (designated 5183892 or 892) as potent inhibitors of HIV-1 gene expression. The compounds were found to suppress HIV-1 gene expression in different cell based assays, reducing accumulation of all viral proteins (Gag, Env, Tat and Rev) tested. Furthermore, a subset of the compounds blocked replication of HIV-1 in PBMCs and inhibited growth of various HIV-1 strains including those resistant to several current therapeutics. Changes in viral protein expression were accompanied by changes in HIV-1 RNA accumulation, all compounds inducing a reduction in US and SS RNA abundance but without significant alterations in MS RNA levels. The compounds had only limited effects on splice site usage that would not account for the loss of Rev and Tat, suggesting that they were affecting Tat/Rev synthesis and/or degradation. Consistent with this hypothesis, addition of the proteasome inhibitor MG132 was found to result in partial restoration of Tat accumulation but not Gag. Parallel examination of the effect of compounds on host RNA splicing revealed that they had very limited effects, suggesting that the response observed was not attributable to a radical alteration in spliceosome function/formation. Of the three, 791 was found to have the least effect on the host, with further analyses determining that, of ~9000 genes surveyed by RNAseq, less than 1% had alterations in alternative exon inclusion of greater than 10%. Together, these findings further establish that compounds can be used to selectively inhibit HIV-1 gene expression at the post-transcriptional level. Further understanding of the mechanism by which these compounds act will help in the refinement of this strategy to control HIV-1 replication.

## Results

### Identification of 791, 833, and 892 as suppressors of HIV-1 protein expression

**Fig. 1:**
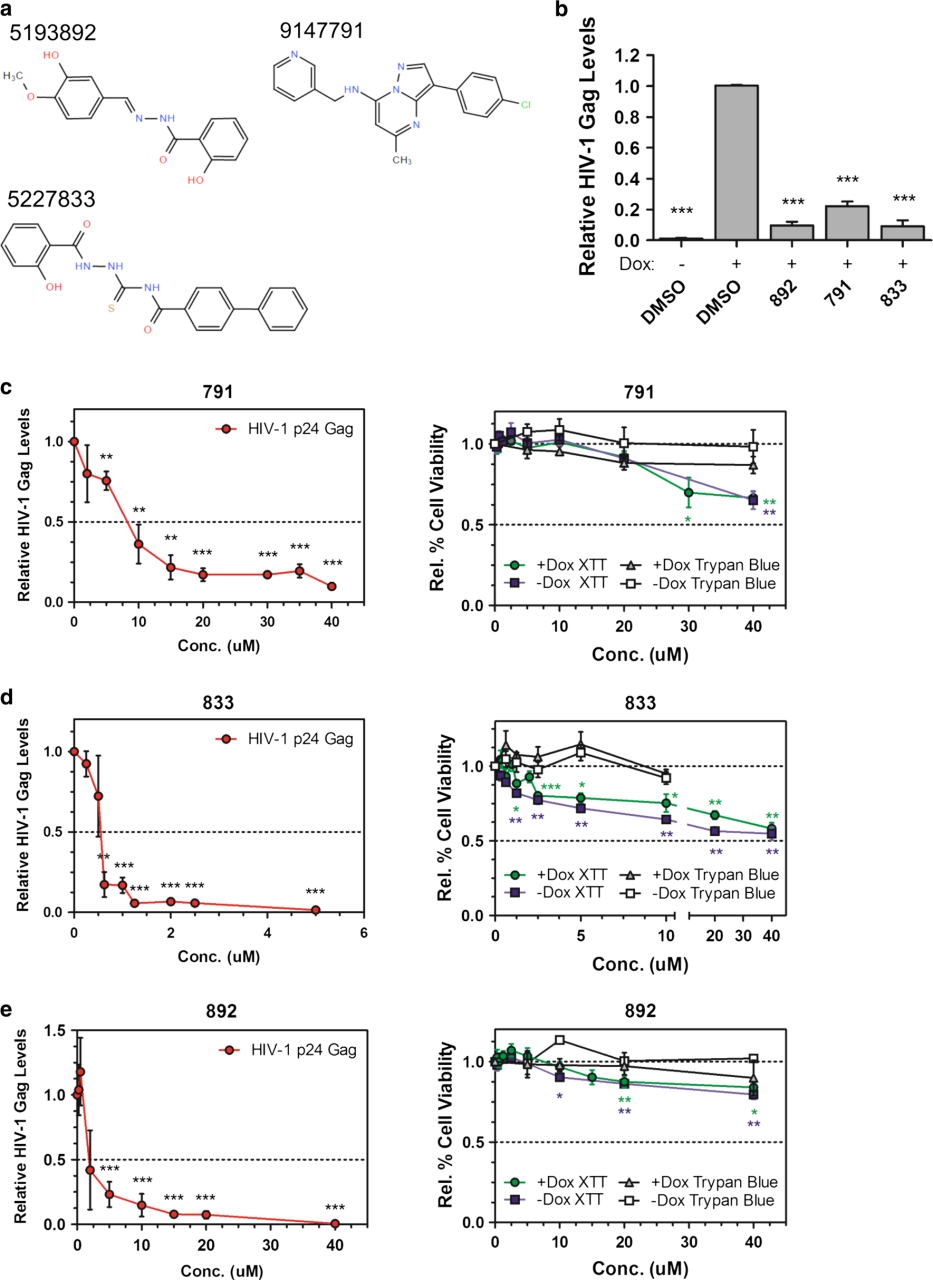
Screen of RNA splicing modulators identifies three potent inhibitors of HIV-1 gene expression. **a** Structures of compounds tested. **b** HeLa HIVrtTA∆*Mls* cells were incubated with 791 (30 µM), 833 (2 µM), or 892 (15 µM) for 24 h in the absence (−) or presence of (+) of Dox and media collected. Effect of compound treatment on HIV-1 virion accumulation in culture supernatant as measured by p24 antigen ELISA and expressed relative to DMSO-treated samples (N ≥ 16, ***p ≤ 0.001). Uninduced, DMSO-treated (DMSO, −Dox) samples were included as negative controls. At left, dose response for 791 (**c**), 833 (**d**), or 892 (**e**) on HIV-1 virion production in culture supernatant was measured by p24 antigen ELISA and expressed relative to p24 Gag levels in DMSO-treated samples (N ≥ 3, *p ≤ 0.05, **p ≤ 0.01, and ***p ≤ 0.001). At right, the effect of the compounds on cell metabolism/viability, at the ranges of concentrations tested, was measured using an XTT assay or Trypan blue exclusion as a readout of viable cells and expressed relative to DMSO-treated samples (N ≥ 3, *p ≤ 0.05, **p ≤ 0.01, and ***p ≤ 0.001). Error bars indicate standard error of the mean (SEM)

The success of digoxin as a potent inhibitor of HIV-1 gene expression, described previously by Wong et al. [14], lead us to screen other small molecular compounds for activity against HIV. Over sixty compounds identified as RNA splicing modulators using the *SMN2* mini-gene reporter system (Dr. Peter Stoilov, University of West Virginia, unpublished) were tested for their ability to inhibit HIV-1 gene expression. As shown in Fig. 1, treatment of HeLa rtTA HIV∆*Mls* cells [12] containing a doxycycline-inducible HIV-1 provirus [18, 19] identified three compounds, designated 791, 833, and 892, that reduced HIV-1 viral production by 80–90% relative to DMSO treatment (+Dox), in the low μM range. The three compounds differed in the number of five and six-numbered rings they contained and lacked a steroid-ring structure like digoxin and other cardiotonic steroids (Fig. 1a). Portions of the 791 structure resemble nucleotide bases, while portions of 892 and 833 structures resemble amido-groups. These compounds were structurally dissimilar to each other and to previously characterized modulators of HIV-1 RNA processing digoxin, 8-azaguanine (8-aza), and 5350150 (150) [13, 14]. Parallel analysis determined that inhibition of HIV-1 replication with compound treatment was dose-dependent. Analysis of compound toxicity by both XTT and Trypan blue exclusion assays determined that for 791 and 833, cell viability was not affected at doses (20 and 10 µM, respectively) required to reduce Gag expression by >80%, although some toxicity was observed at higher doses. Compound 833 showed some reduction in mitochondrial function as indicated by XTT assays but no change in Trypan blue exclusion up to 10 µM whereas a 90% reduction in Gag expression was achieved at 1.5 µM (Fig. 1c–e; Table 1). No difference in compound toxicity was observed in the absence or presence of HIV-1 gene expression (Fig. 1c–e, ∓Dox). Both 791 and 833 maintained their inhibition of HIV-1 gene expression in the context of CD4^+^ SupT1 cells (Additional file 1: Figure S1).

**Table 1:** Effect of compounds on HIV-1 gene expression and cell viability

	HeLa			24STNLESG		CEM-GXR			PBMC	
	IC_50_ (μM)	CC_50_ XTT (μM)	CC_50_ Trypan blue (μM)	IC_50_	CC_50_ XTT	IC_50_	CC_50_ FSC/SSC (μM)	CC_50_ ViaCount (μM)	IC_50_ (μM)	CC_50_ Trypan blue (μM)
791	8.2	>40	>40	26 μM	>60 μM	4.5 μM	>12	24	1.5	> 10
833	0.6	≥40	>10	3.5 μM	>20 μM	1.0 μM	>5	>5	1.6	3.0
892	1.8	>40	>40	nd	nd	n/a	31	60	2.0	4.5

The concentration of compounds that inhibit HIV gene expression by 50% (IC_50_) and the concentration that decreases cellular toxicity to 50% (CC_50_) relative to DMSO-treated cells are listed. Compound toxicity was measured by either a cellular metabolism assay (XTT). trypan blue exclusion, fluorescence-activated cell sorting (FACS) or Guava ViaCount assay. For the CEM-GXR cells, the CC_50_ was based on the compound toxicity tn cells infected with a Clade B HIV-1 strain, while uninfected calls were used for the ViaCount assay. *FSC* forward scatter, *SSC* side scatter, *nd* not done. *n/a* not available

### Compounds inhibit HIV-1 replication in PBMCs and are effective against drug resistant forms of HIV-1

**Fig. 2:**
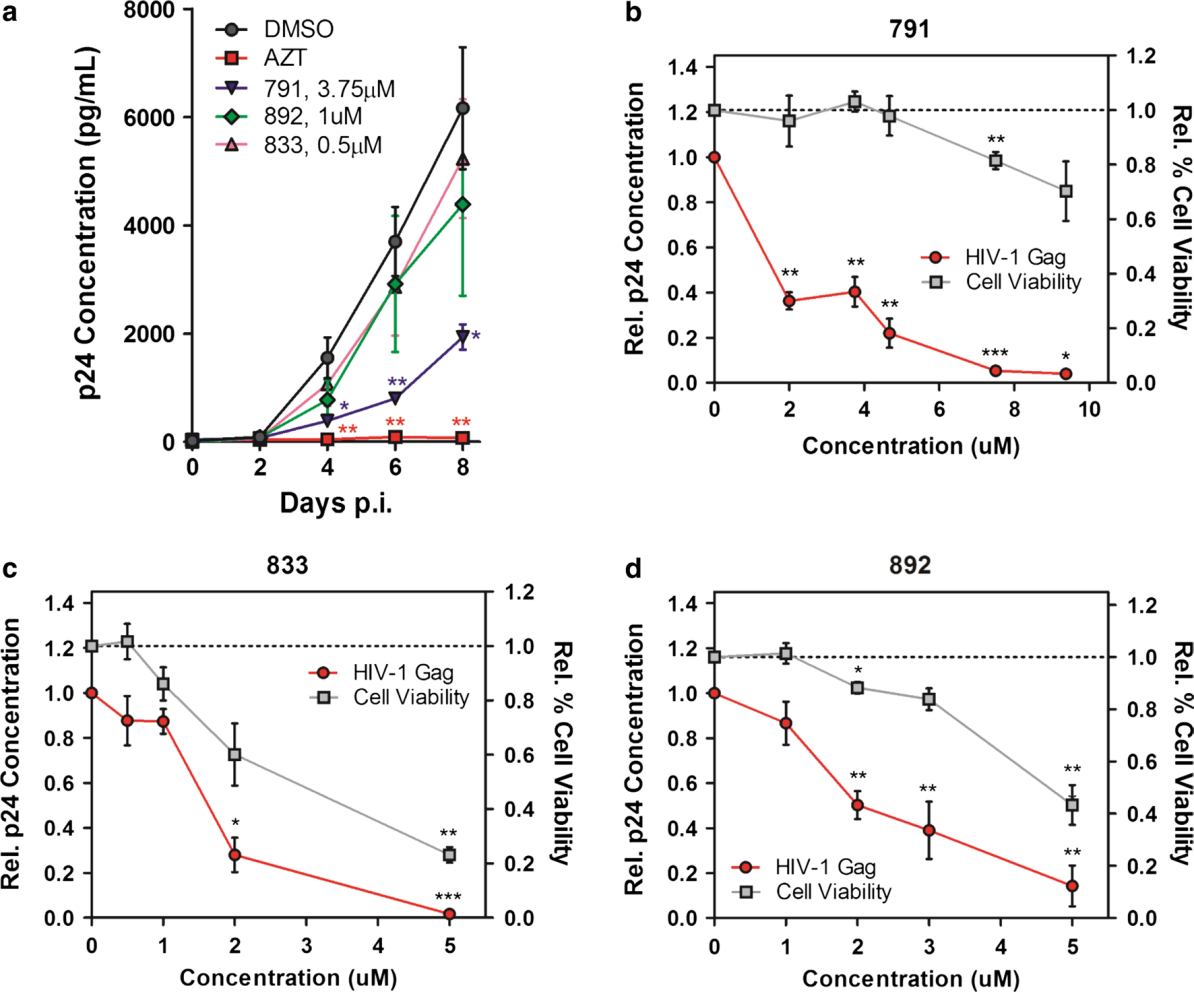
791 inhibits HIV-1 replication in PBMCs with limited toxicity. **a** Average data of HIV-1 BaL virus replication in three different donors over a period of 8 days post-infection (p.i.,) as measured by p24 antigen ELISA (N = 4, 3 donors). PBMCs were infected with HIV-1 BaL (MOI < 0.01) and treated on days 0 and 4 post infection with DMSO, AZT (3.74 μM), 791, 833, or 892 at the concentrations indicated. *Error bars* indicate standard error of the mean (SEM). The effect of increasing concentrations of 791 (**b**), 833 (**c**), or 892 (**d**) on HIV-1 BaL virion production in PBMCs. Following infection, indicated doses of compound were added, media harvested after 6 days, virus replication measured by p24 antigen ELISA and expressed relative to p24 Gag levels in DMSO-treated cultures (N ≥ 3, *p ≤ 0.05, **p ≤ 0.01, and ***p ≤ 0.001). The effect of the compounds on cell viability was measured by trypan blue exclusion as a percentage of total cells and expressed relative to percent cell viability with DMSO-treatment

The ability of the compounds to potently inhibit HIV-1 gene expression in the context of the cell lines tested raised the question as to whether they would have similar effects on replicating virus in primary cells. Consequently, we tested the ability of 791, 833, or 892 to inhibit HIV-1 BaL replication in peripheral blood mononuclear cells (PBMCs) from healthy donors. PBMCs were activated for three days prior to infection with HIV-1 BaL (multiplicity of infection, MOI < 0.01) and treatment with DMSO, 791, 833, or 892. Cell culture medium from compound-treated cells was sampled every 2 days to measure the effect of compound treatment on virus production and cell viability. Inhibition of HIV-1 virus production in PBMCs by 791 was achieved in at least three independent experiments using cells from 2–4 different donors at doses (<5 µM) that did not affect cell viability (representative data shown in Fig. 2). In contrast, 833 and 892 displayed toxicity at concentrations required to block HIV-1 replication. Therefore, 791 inhibited HIV-1 replication in a mixed cell population even under in vitro HIV infection conditions where cell infection rates are substantially higher than in HIV^+^ patients. Furthermore, 791 maintains inhibitory activity in primary cells against replication-competent HIV-1 at similar or lower concentrations than needed in HeLa cell lines, demonstrating that the compound is active at low μM concentrations in a relevant context.

**Fig. 3:**
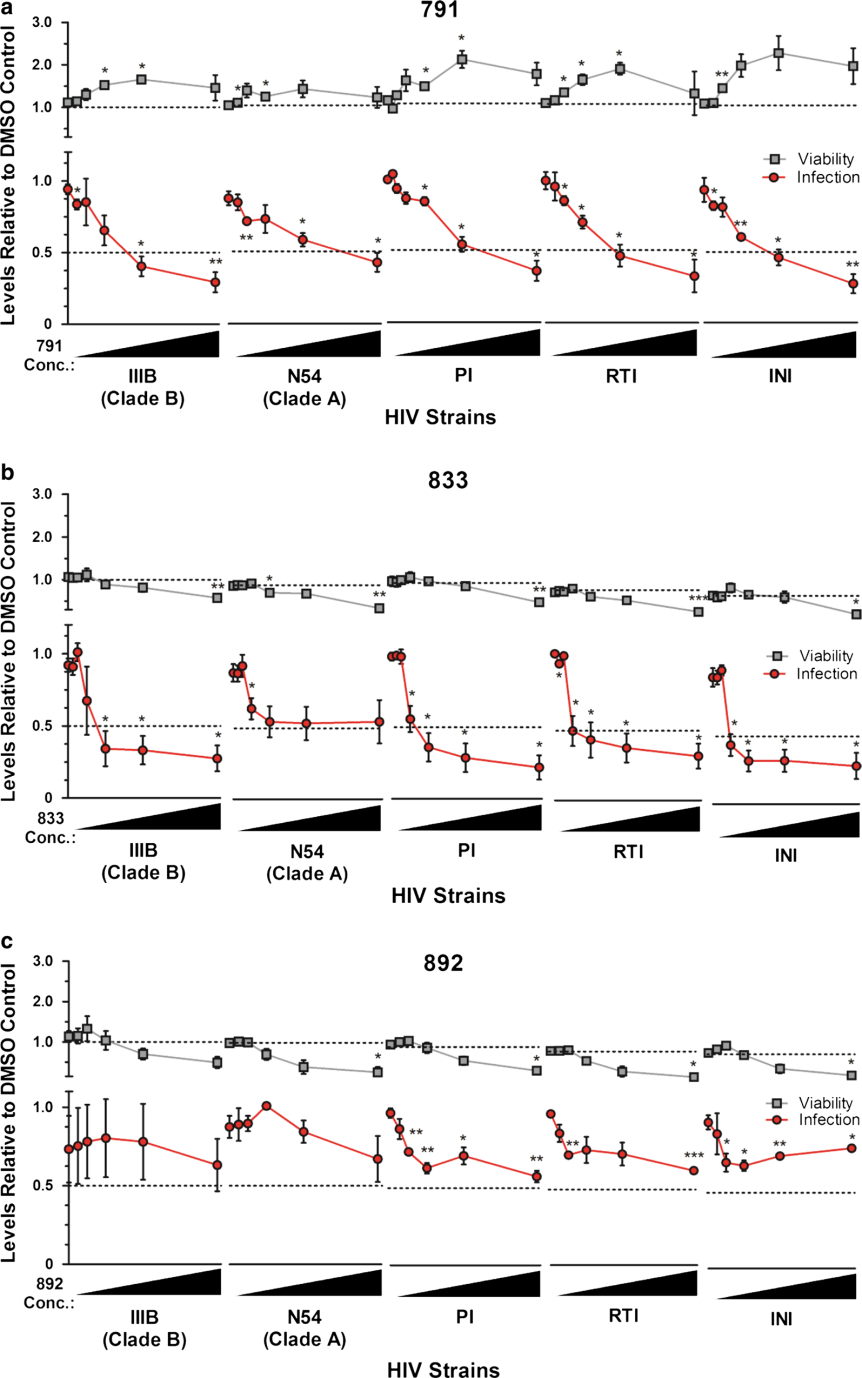
791 and 833 inhibit replication of multiple HIV-1 variants. To assess the ability of compounds to inhibit replication of different HIV-1 strains and viral variants having resistance to HIV-1 inhibitors, CEM-GXR cells, containing an exogenous Tat-driven LTR-GFP expression cassette, were infected with clade A (97USSN54 (N54)) or clade B (IIIB) strains of HIV-1 or pNL4-3 virus having mutations conferring resistance to protease inhibitors (PRI, strain 2948), reverse transcriptase inhibitors (RTI, strain E00443)), or integrase inhibitors (INI, strain 11845). See Additional file 3: Table 1 for a full description of the drug resistant viruses used. Cell cultures containing serial dilutions of the molecule in the final concentrations of **a** 0.725–46.87 μM for 791, **b** 0.156–5 μM for 833, or **c** 1.95–62.5 μM for 892. Antiviral activity was evaluated measuring frequency of HIV-1 infected (GFP positive) cells by flow cytometric analysis. For viable cell counts, the gate in a flow cytometer was set to cover 95% of the freshly passaged uninfected CEM-GXR cell or ViaCount™ was used

As a further test of the compounds, we examined their ability to inhibit replication of clade A (HIV-1 N54) and B (HIV-1 IIIB) viruses as well as strains resistant to reverse transcriptase, protease, or integrase inhibitors. As shown in Fig. 3, both 833 and 791 yielded significant reductions in HIV-1 replication of all viruses tested with limited effects on cell viability (as assessed by forward scatter/side scatter (FS/SS) and ViaCount, Fig. 3; Table 1 and Additional file 2: Figure S7). 892 displayed only modest inhibitory activity against all viruses at the doses examined.

### 791, 833, and 892 block expression of HIV-1 Gag, Env, Tat and Rev

**Fig. 4:**
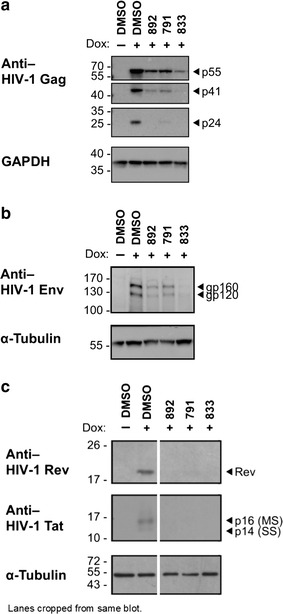
Compound treatment dramatically decreases the expression of HIV-1 structural and regulatory proteins. HeLa HIVrtTA∆*Mls* cells were incubated with DMSO (control), 791 (30 µM), 833 (2 µM), or 892 (15 µM) for 24 h in the absence (−) or presence of (+) of Dox. Cells were subsequently harvested and shown are representative blots showing the effect of the compounds on HIV-1 **a** Gag protein and **b** Env protein expression relative to GAPDH or α-tubulin expression as loading controls (SDS-PAGE, N ≥ 3). Images showing p55, p41, and p24 expression were cropped from same blot visualized at different exposure times due to difference in abundance of these isoforms. **c** Representative blots showing the effect of the compounds on HIV-1 Rev and Tat protein expression relative to α-tubulin expression as loading control (SDS-PAGE, N ≥ 3). For the blot shown, lanes were cropped from the same blot to show compound-treated lanes adjacent to DMSO-treated control lanes

To understand the basis for the inhibition of virus production, we examined the effect of the compounds on expression of multiple viral proteins. Following treatment of HeLa rtTA HIV∆*Mls* cells and doxycycline induction for 24 h, cell lysates were harvested and blots probed with antibodies to the viral structural proteins Gag and Env, as well as regulatory proteins Rev and Tat. Representative western blots from at least three independent experiments are shown (Fig. 4). All compounds reduced the levels of p55, p41, and p24 Gag proteins and gp160 and gp120 Env proteins relative to DMSO alone (Fig. 4a, b). The effect of the compounds on viral regulatory proteins, however, is very different from that observed with previously characterized HIV-1 inhibitors (Fig. 4c) [12–14]. All three compounds resulted in significant reduction in accumulation of both Tat and Rev. Together, these results suggest that 791, 833, and 892 inhibit HIV-1 protein expression in vitro by blocking expression of both early (Rev, Tat) and late (Gag, Env) HIV-1 proteins (Fig. 4).

### 791, 833, and 892 reduce HIV-1 US and SS RNA but not MS RNA accumulation

**Fig. 5:**
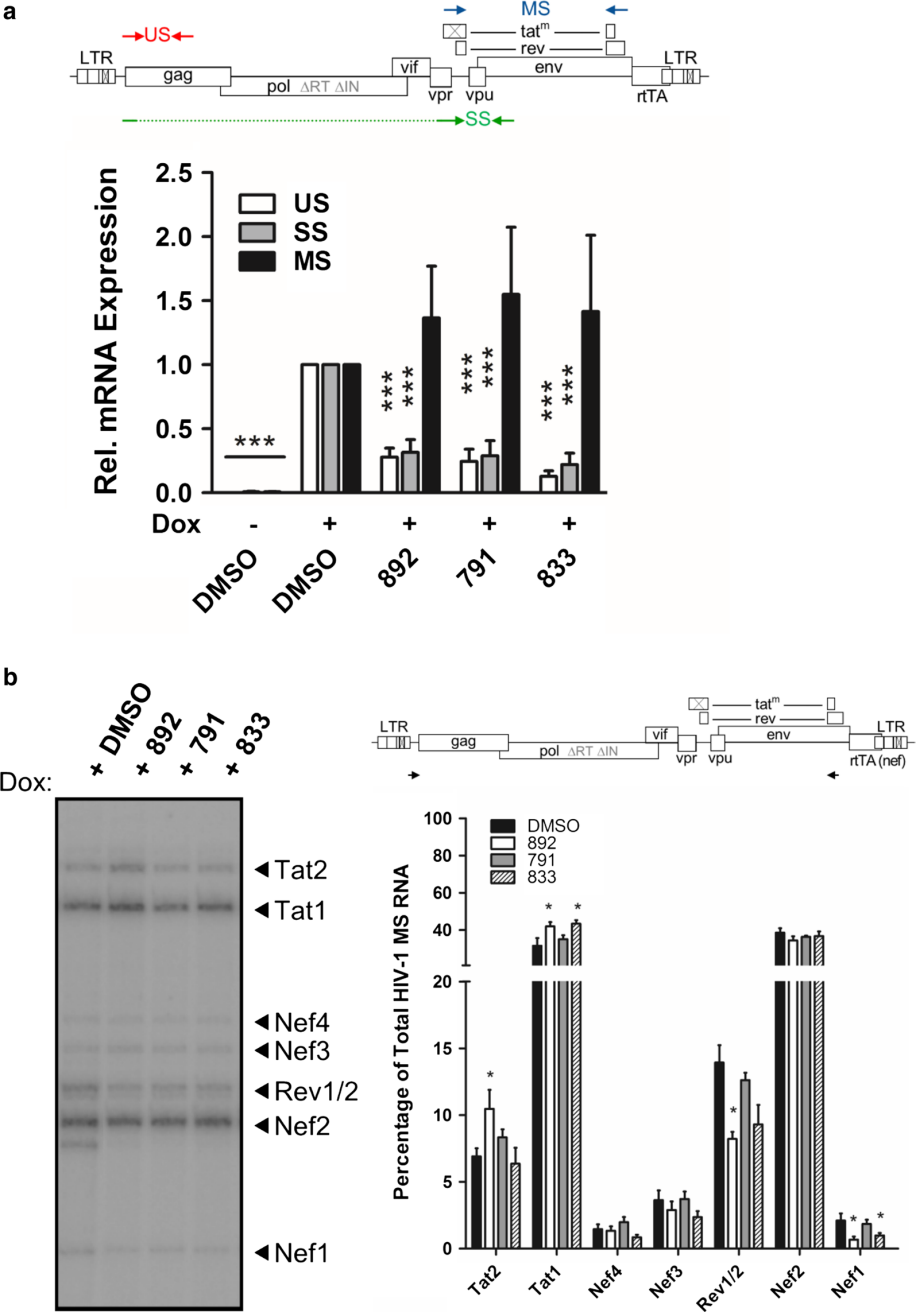
The compounds decrease the levels of HIV-1 US and SS RNAs but do not dramatically alter splice site usage. HeLa HIVrtTA∆*Mls* cells were incubated with 791 (30 µM), 833 (2 µM), or 892 (15 µM) for 24 h in the absence (−) or presence of (+) of Dox, then cells collected and total RNA extracted. **a** *Top* schematic of HIV-1 genome with the positions of the forward and reverse primers used for qRT-PCR analysis indicated by the *arrows*. *US* unspliced, *SS* singly spliced and *MS* multiply spliced. *Bottom*, quantification of viral mRNA levels in compound-treated samples were normalized to β-actin and the mean mRNA levels expressed relative to DMSO-treatment (N ≥ 7, **p ≤ 0.01, and ***p ≤ 0.001). *Error bars* indicate standard error of the mean (SEM). **b** *Top*, schematic of HIV-1 genome with the positions of the forward and reverse primers used to amplify the 1.8 kb class of HIV-1 RNAs indicated by the *arrows*. *Left* representative RT-PCR gel with HIV-1 MS isoforms labelled on the right according to Purcell and Martin, 1993 (N ≥ 3). *Right* quantification of PCR products was performed by densiometry analysis with the level of each isoform expressed as the mean percentage of the total density of all RNA species within the sample (N ≥ 7, **p ≤ 0.01, and ***p ≤ 0.001. *Error bars* indicate standard error of the mean (SEM)

To determine whether the dramatic loss of viral proteins is accompanied by changes in viral mRNA levels, the effect of compound treatment on the abundance of HIV-1 RNA classes was examined by qRT-PCR. Total RNA was isolated from DMSO- or compound-treated HeLa rtTA HIV∆*Mls* cells, and qRTPCR performed using forward and reverse primers specific to β-actin (internal control for normalization) as well as HIV-1 unspliced (US), singly-spliced (SS), and multiply spliced (MS) RNAs. Analysis of HIV-1 RNA abundance revealed that the compounds reduced levels of HIV-1 US and SS RNAs with no significant changes in levels of MS RNA relative to DMSO alone. Uninduced, DMSO-treated cells showed no viral RNA expression, as expected (Fig. 5a). This data correlated with the reduced levels of Gag, Env, and p14 Tat (Fig. 4) since these proteins are encoded by HIV-1 US and SS RNAs. However, the imbalance in viral RNA classes suggested that the compounds may be altering viral RNA splicing, a critical step in HIV-1 replication that relies heavily on regulation of splicing involving many cellular factors [1].

Given that HIV-1 MS RNA abundance is unaffected by compound treatment but MS-encoded proteins, Rev and Tat, are reduced, the compounds could be inducing changes in splice site usage to alter the levels of MS RNA splice variants coding for these proteins. Hence, we performed RT-PCR for MS RNAs to assess whether the compounds altered usage of specific splice sites within this class of RNAs. Although the HIV-1 proviral genome in HeLa rtTA HIV∆*Mls* cells contains modifications, it recapitulates the splicing events of HIV-1 pre-RNA, so that the levels of most MS RNA isoforms (less abundant isoforms are below the limit of detection) can be analyzed using this method [20]. Amplified products were visualized and the levels of HIV-1 MS RNA isoforms quantified by densiometric analysis and designated according to size. (see Additional file 4: Figure S2 for a description of the splice products indicated). No significant changes in splice site usage were observed upon 791 treatment relative to control (DMSO, +Dox) (Fig. 5b). In contrast, 892 and 833 treatment caused a ~30% decrease in levels of Rev1/2 and Nef RNAs and slightly increased (<10%) Tat1 and Tat2 RNAs, relative to DMSO. Such minor alterations in splice utilization coupled with the lack of effect on MS RNA levels suggests that the loss of HIV-1 regulatory proteins upon compound treatment cannot be attributed to a marked reduction of viral MS RNAs encoding these proteins. Consequently, the compounds are more likely to interfere with the translation of these MS RNAs or alter the stability of the proteins synthesized.

### Compounds inhibit cytoplasmic accumulation of HIV-1 US RNA consistent with loss of Rev function

**Fig. 6:**
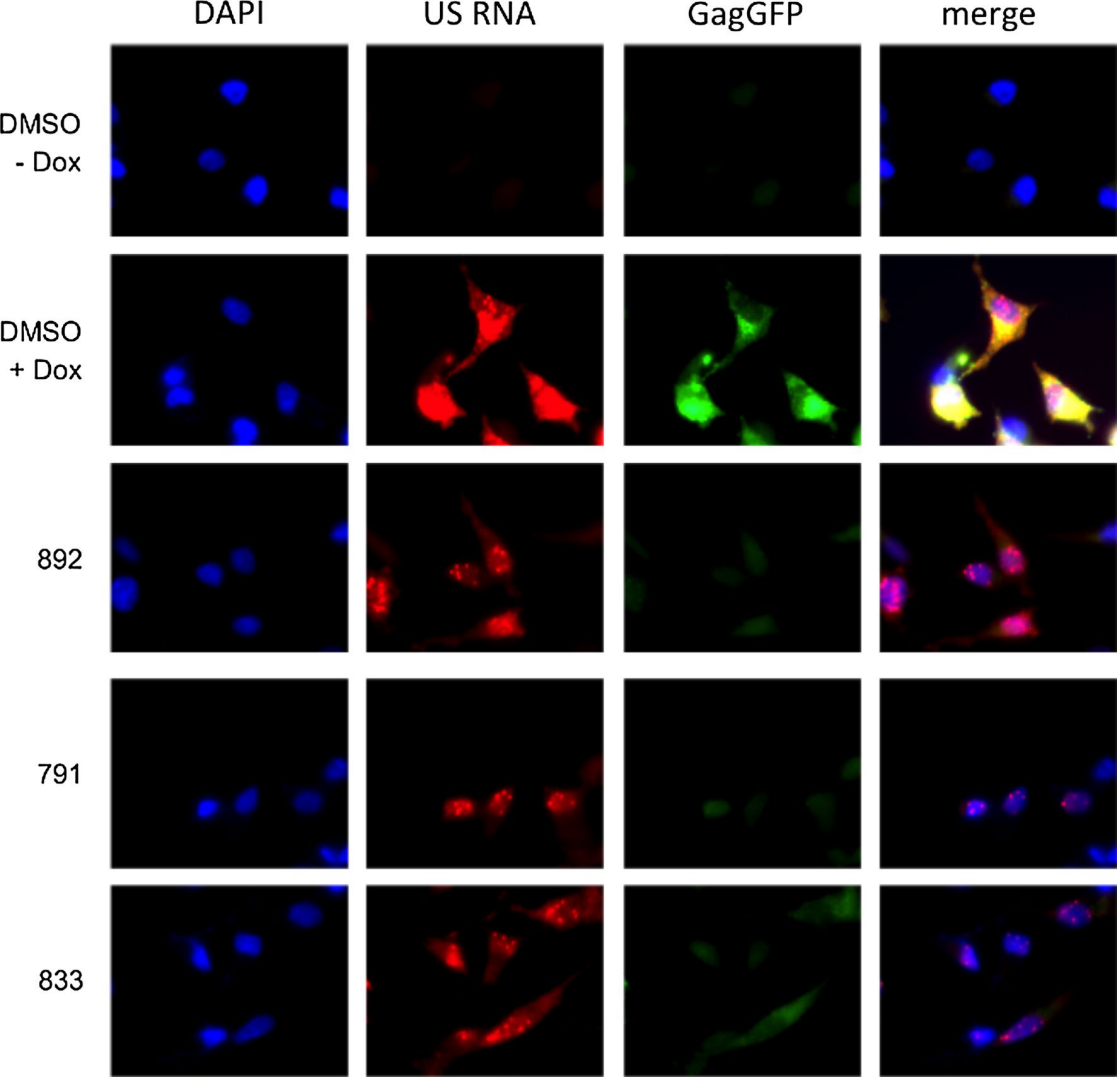
791, 833, and 892 inhibit cytoplasmic accumulation of HIV-1 US RNA. HeLa rtTA HIV GagGFP C7 cells were treated with 791 (30 µM), 833 (2 µM), or 892 (15 µM) for 4 h prior to Dox induction. Cells were fixed 24 h after Dox addition and processed for FISH using probes to HIV-1 US RNA. Representative fluorescent in situ hybridization images of cells treated with DMSO or the indicated compounds (N ≥ 3). Cells were viewed at 630X (oil immersion) magnification. Images are cropped to show a representative field of view

Since compound treatment resulted in loss of HIV-1 MS-encoded regulatory proteins Rev and Tat, but had no appreciable effect on the abundance or splice site usage within MS RNA, we hypothesized that the compounds may inhibit HIV-1 gene expression by perturbing Rev-mediated viral RNA transport, protein synthesis, or protein stability. To assess the effect of the compounds on the Rev-dependent export of incompletely spliced viral RNA, the subcellular localization of HIV-1 US RNA and Gag was examined by fluorescent in situ hybridization (FISH). Since the compounds induce loss of Rev protein (Fig. 4c), it was likely that HIV-1 US RNA would not be exported to the cytoplasm for subsequent virus particle assembly and translation of viral structural proteins [21, 22]. To determine if this was the case, HeLa rtTA HIV GagGFP (C7) cells, which express a GagGFP fusion protein, were treated with DMSO or compounds as described above and localization of HIV-1 US RNA assessed by FISH. Studies confirmed that this cell line had similar response to compound addition as that detailed above (see Additional file 5: Figure S3). Induction of HIV-1 gene expression (DMSO, +Dox) results in US RNA localization in both the nucleus and cytoplasmic region with strong GagGFP expression throughout the cell (Fig. 6). Co-localization of viral US RNA and GagGFP is indicated by the merged signal (yellow). In contrast, treatment with 833, 791, or 892 prevented cytoplasmic accumulation of HIV-1 US RNA and reduced GagGFP levels relative to DMSO treatment (N ≥ 3). No US RNA and GagGFP expression was detected in uninduced cells, as expected. The effect of the compounds on HIV-1 US RNA and GagGFP expression is consistent with US RNA abundance and Gag protein expression measured by qRT-PCR and SDS-PAGE, respectively. Furthermore, the nuclear retention of US RNA upon compound treatment is consistent with the loss of Rev protein observed by western blotting (Fig. 4). These results suggest that the compounds prevent the early to late transition in HIV-1 gene expression.

### 791 and 833 do not affect total protein synthesis

**Fig. 7:**
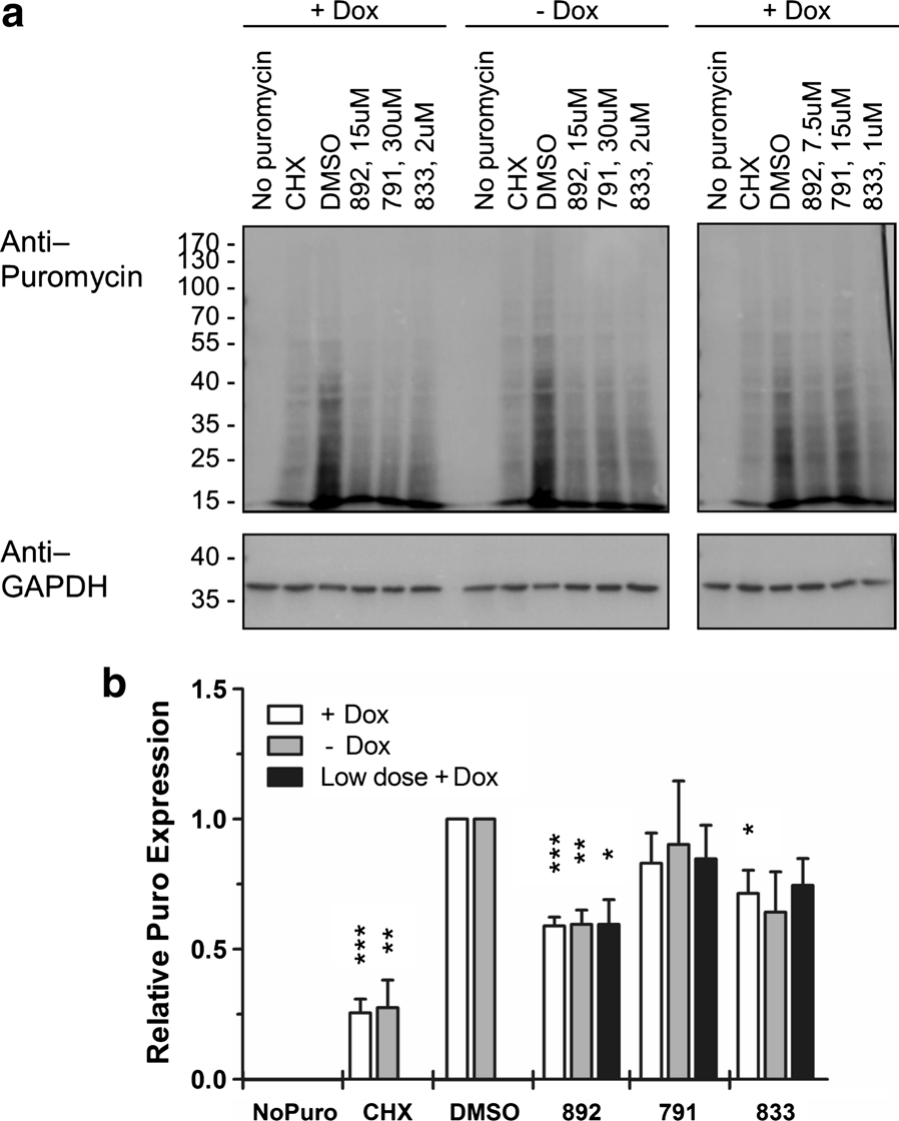
791 and 833 have limited effect total protein synthesis. HeLa HIVrtTA∆*Mls* cells were incubated with 791, 833, or 892 for 24 h at doses indicated in the absence (−) or presence of (+) of Dox. Puromycin was added 30 min prior to harvest to measure total protein synthesis using SUnSET. **a** Representative blot showing the effect of the compounds on protein synthesis by puromycin labelling of nascent polypeptides (N ≥ 4). Samples not incubated with puromycin (No Puro) or treated with cycloheximide (CHX) to block translation, served as negative controls. **b** Quantification of protein synthesis in the presence of the compounds was measured by the volume intensity in each lane normalized to GAPDH intensity and expressed relative to the DMSO-treatment (N ≥ 4, *p ≤ 0.05, **p ≤ 0.01, ***p ≤ 0.001). *Error bars* indicate standard error of the mean (SEM)

The loss of expression of all HIV-1 proteins tested raised the possibility that some or all of the compounds were acting as general inhibitors of protein synthesis. To address this hypothesis, the effect of compound treatment on protein synthesis was measured by surface sensing of translation (SUnSET) [23]. This nonradioactive method uses puromycin, a structural analog of aminoacyl tRNAs produced by *Streptomyces alboniger*, to monitor protein synthesis since it becomes incorporated into nascent peptides when inducing chain termination. Consequently, new protein synthesis was measured by addition of puromycin to the media 30 min prior to harvest and measurement of incorporation by western blot using a monoclonal antibody to puromycin. Analysis of blots in the presence and absence of HIV-1 gene expression (∓Dox) indicated that both 791 and 833 had little or no effect while 892 resulted in a small reduction (~25%) in protein synthesis relative to DMSO at doses that significantly reduced HIV-1 protein expression (Fig. 7). In contrast, cells treated with cycloheximide (CHX), an inhibitor of translation elongation, or without puromycin (No puro), had little or no signal, respectively, confirming that signals detected were newly synthesized puromycin-tagged polypeptides.

### 791, 833, and 892 alter Tat protein synthesis and/or stability

**Fig. 8:**
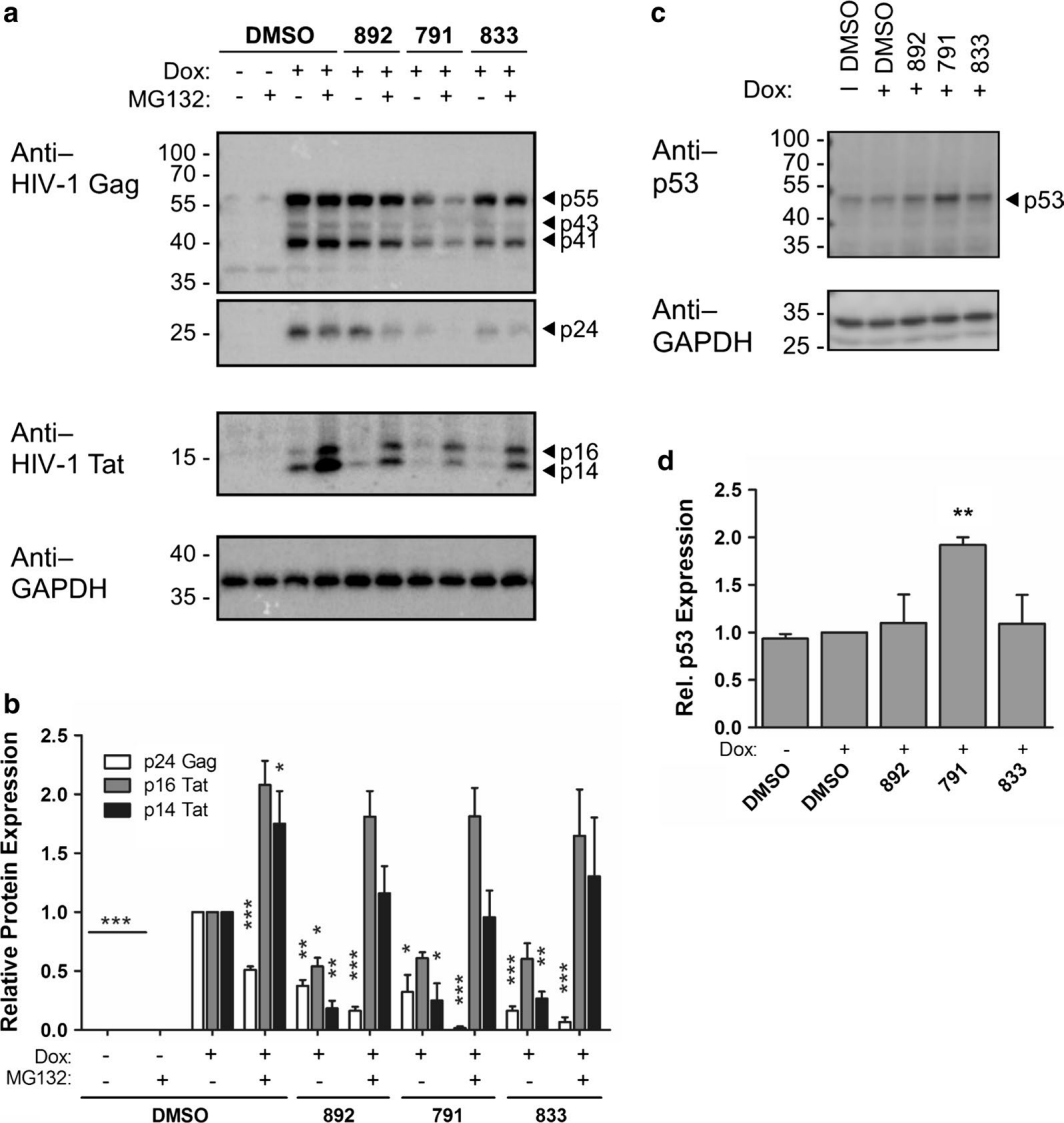
HIV-1 Tat expression can be rescued with proteasome inhibition by MG132. HeLa HIVrtTA∆*Mls* cells were incubated with 791 (30 µM), 833 (2 µM), or 892 (15 µM) for 24 h in the absence (−) or presence of (+) of Dox. Eight hours prior to harvest, MG132 was added as indicated to a subset of the samples. **a** Representative blot showing effect of the compounds on HIV-1 Gag and Tat expression in the presence or absence of proteasome inhibitor MG132, relative to DMSO treatment. GAPDH serves as loading control. **b** Summary of band intensities of HIV-1 p24 Gag and p14 and p16 Tat with each treatment relative to that of the DMSO control normalized to the corresponding GAPDH bands (N ≥ 3). *Error bars* depict standard error of the mean and *, **, and *** indicate *P* values ≤ 0.05, 0.01, and 0.001, respectively. **c**, **d** To assess effect of compound addition on p53 expression, lysates were prepared from cells treated with 791 (30 µM), 833 (2 µM), or 892 (15 µM) and blots were probed for p53 and GAPDH. Shown are (**c**) representative blot of results and **d** summary of quantitation of p53 expression after normalization using GAPDH

Given the absence of an effect on total protein synthesis that could account for the loss of Tat or Rev expression, it was of interest to examine whether the compounds had a more direct effect on HIV-1 protein synthesis or stability. Initial attempts to evaluate whether the compounds directly affected Tat protein decay did not detect any measurable changes in the kinetics of Tat protein loss (see Additional file 6: Figure S4). To determine whether the change in Tat expression could be attributed to lack of synthesis or enhanced turnover, we tested the effect of MG132 (a proteasome inhibitor) treatment on Tat and Gag protein levels in the presence and absence of compound. As shown in Fig. 8a, b, addition of MG132 resulted in significant accumulation of both Tat isoforms in the presence of 791, 833, or 892. In contrast, MG132 did not restore production of HIV-1 Gag to any significant level relative to that seen in the absence of MG132. The ability to restore Tat protein accumulation with MG132 confirms that Tat protein synthesis is occurring in the presence of the compounds. To assess whether the compounds were acting in a manner similar to another recently described small molecule modulator of Tat stability [24], we examined changes in cellular p53 levels upon compound addition. In contrast to curcumin, which reduces both Tat and p53 levels [24], addition of 833 or 892 had no effect on p53 expression while 791 increased the levels of this host factor by approximately twofold (Fig. 8c, d).

### 791, 833, and 892 did not significantly affect cellular RNA alternative splicing

**Fig. 9:**
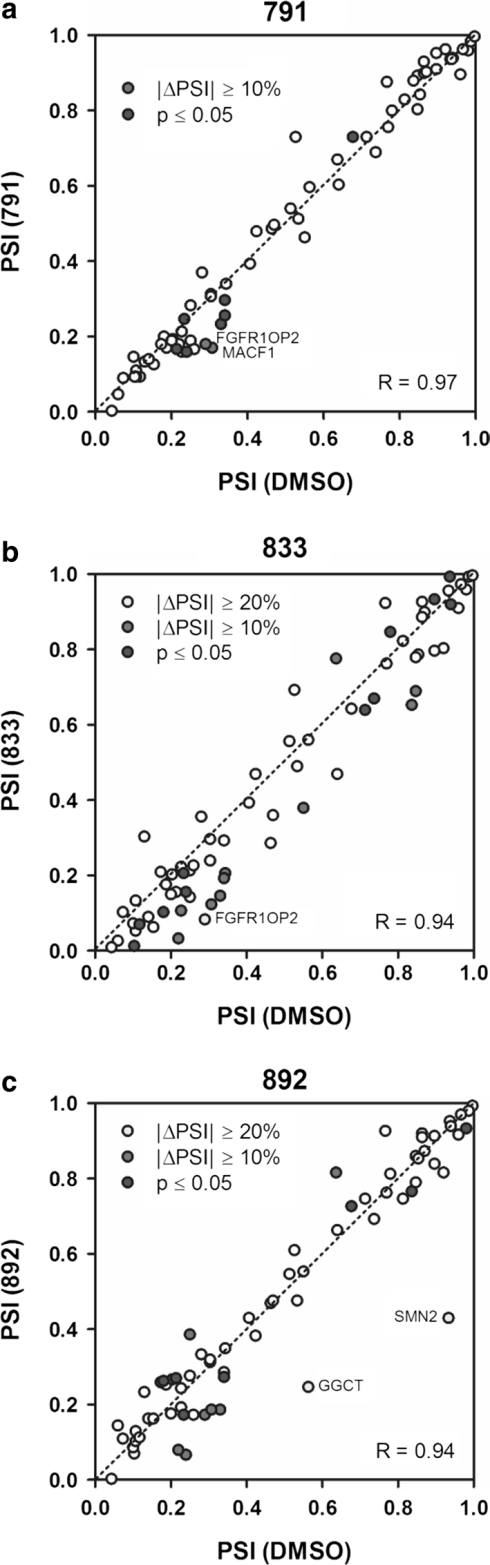
791, 833, and 892 have limited effects on cellular alternative splicing events. HeLa HIVrtTA∆*Mls* cells were incubated with **a** 791 (30 µM), **b** 833 (2 µM), or **c** 892 (15 µM) for 24 h in the presence of Dox. Total RNA was harvested and used in RT-PCR assays to monitor changes in 73 host genes known to undergo alternative splicing. Mean alternative splicing changes (PSI, percent spliced in) were plotted comparing DMSO and compound treatment (N = 3, RT-PCR). *Diagonal dotted line* no difference between treatments. *Dots above/below the diagonal* increased/decreased exon inclusion. |ΔPSI| ≥ 10 and 20% are indicated as *red* and *yellow dots* (labelled), respectively. Statistically significant alternative splicing changes with |ΔPSI| ≤10% are indicated by the gray dots (Student’s *t* test, two-tailed). *Error bars* not shown. Pearson correlations (R values) are shown. See Additional files 7, 8, 9: Tables S2, S3, S4 for a complete description of the data obtained

Given the dramatic shift in HIV-1 RNA accumulation, it was of interest to determine to what extent alternative splicing of the host cell RNAs was altered. As an initial measure, we evaluated the effect of compound treatment on alternative splicing of 73 select endogenous transcripts (see Additional files 7, 8, 9: Tables S2, S3, S4) by RT-PCR using RNA isolated from DMSO and compound-treated HeLa rtTA HIV∆*Mls* cells and quantitated by capillary electrophoresis of the amplicons generated. The ‘percent spliced in’ or PSI in annotated cassette exons was compared to DMSO treatment (Fig. 9, Additional files 7, 8, 9: Tables S2, S3, S4). Changes in alternative splicing of endogenous genes/transcripts with |ΔPSI| ≥ 10 and 20% are represented as red and yellow dots, respectively, and a subset of these genes are labelled next to their respective data points. Treatment with 791 showed no appreciable changes in alternative splicing of the examined events as most events fell along the theoretical diagonal dotted line depicting no difference between compound and DMSO treatments (Pearson correlation coefficient, R = 0.97). Both 833 and 892 increased and decreased exon inclusion in a small subset of transcripts as indicated by the points falling above or below the diagonal line, but overall showed a strong correlation with DMSO treatment (R = 0.94). Of note, three alternatively spliced exons, *fgfr1op2*, *macf1*, and *gm130/golga2* were affected by all three compounds.

**Fig. 10:**
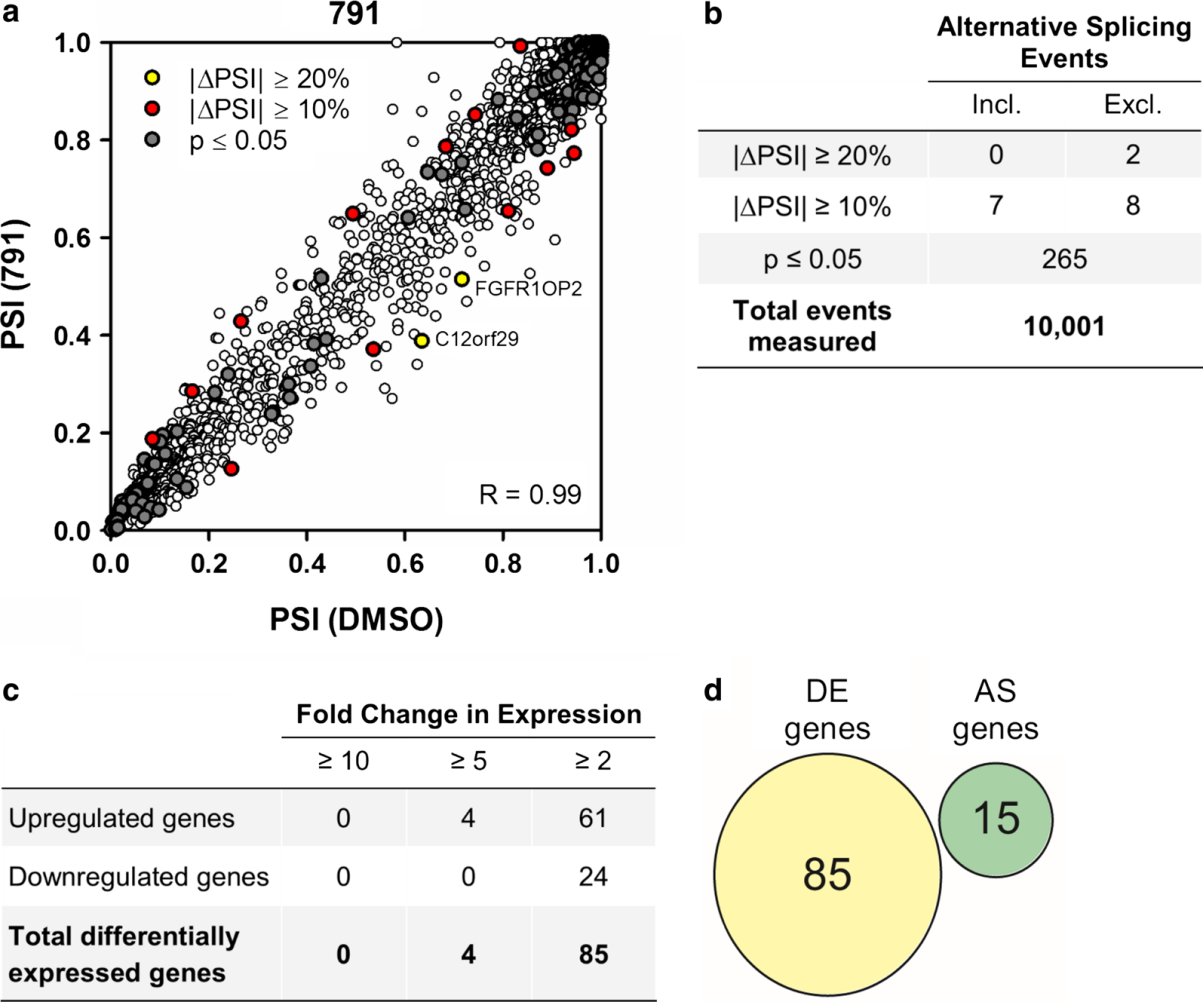
791 does not appreciably alter cellular gene expression or alternative splicing events. HeLa HIVrtTA∆*Mls* cells were incubated with 791 (30 µM) for 24 h. Cells were subsequently harvested for total RNA isolation and samples used for cDNA library preparation and Illumina sequencing. **a** Mean alternative splicing changes (PSI or percent spliced in) were plotted comparing DMSO and compound treatment (N = 2, RNA-seq). |ΔPSI| ≥ 10% and 20% are represented as red and yellow dots, respectively. AS genes with exon inclusion/exclusion ≥20% are labelled. Statistically significant alternative splicing changes with |ΔPSI| ≤ 10% are indicated by the gray dots (Student’s t test, two-tailed). *Error bars* not shown. **b** Summary of altered exon inclusion or exclusion (Incl. or Excl.) with compound treatment (RNAseq, N = 2). **c** Differentially expressed (DE) genes described as cRPKM fold change ≥2 or ≤0.5 with compound treatment relative to DMSO treatment (p ≤ 0.05, 11,406 genes, N = 2). Fold change distribution of differentially expressed genes based on compound treatment relative to DMSO treatment within the RNAseq dataset (p ≤ 0.05, 1020 genes, N = 2). **d** Venn diagrams comparing DE and AS (|ΔPSI| ≥ 10%) events with 791 (N = 2). See Additional files 10, 11: Tables S5 and S6 for a complete description of the data obtained

To gain a more comprehensive evaluation of the effect of 791 on host RNA alternative splicing and expression in an unbiased fashion, paired-end RNAseq was performed on RNA isolated from DMSO and 791 treated HeLa rtTA HIV∆*Mls* cells (Fig. 10, Additional files 10, 11: Tables S5, S6). 791 was of particular interest given its consistent anti-HIV-1 activity in all assays (see Figs. 2, 3) and more limited effect on cell growth upon extended culture with cells (see Additional file 12: Figure S5). To calculate altered splicing events in response to 791 treatment, the PSI in annotated cassette exons was determined and compared to DMSO treatment. Based on the analysis of biological duplicates of the ~10,000 alternatively spliced events detected, 791 treatment resulted in very few altered splicing events (2 AS events with exon inclusion/exclusion ≥20% out of ~10,000 events) and correlated well (R = 0.99) with values in DMSO treated samples (Fig. 10a, b). The patterns of alternative splicing changes observed by RNAseq were consistent with data from the subset of AS events measured by the RT-PCR outlined previously (Fig. 9). These results indicate that 791 did not significantly perturb cellular alternative splicing and suggest that its inhibitory effect is selective to processes involved in HIV-1 gene expression.

To determine whether 791 induced changes in gene expression, alterations in mRNA abundance were examined and compared with changes in alternative RNA splicing. The differential expression level of genes with DMSO or 791 treatment was quantified as corrected reads per kilobase of exon model per million mapped (cRPKM) reads. The expression cutoff was a cRPKM value of 0.5, corresponding to ≥10 reads that uniquely mapped to a single genomic locus. Genes were described as differentially expressed (DE) if the cRPKM fold change was ≥2 or ≤0.5. Of 11,406 total genes examined, relatively few DE genes were detected following compound treatment (Fig. 10c, Additional file 11: Table S6). In fact, 791 treatment changed only 0.46% of all genes analyzed relative to DMSO treatment. Of the genes whose expression levels were altered, *trib3*, a putative protein kinase, increased ninefold with 791 treatment relative to DMSO addition (N = 2; see Additional file 11: Table S6). Comparison of changes in gene expression (GE) versus alternative splicing (AS) in response to 791 addition revealed no overlap in the genes being affected (Fig. 10d).

**Fig. 11:**
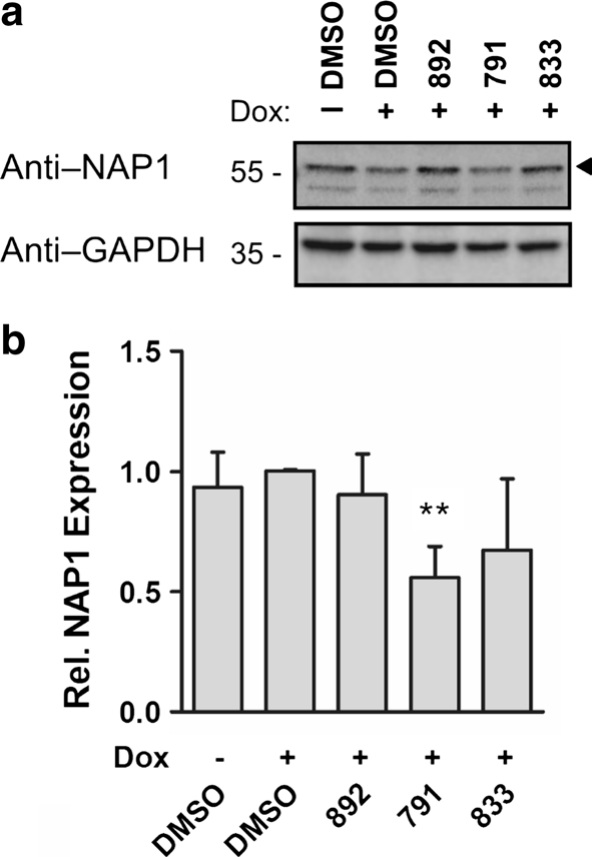
791 addition reduces levels of the protein chaperone Nap1. HeLa HIVrtTA∆*Mls* cells were incubated with 791 (30 µM), 833 (2 µM), or 892 (15 µM) for 24 h in the absence (−) or presence of (+) of Dox. Cell lysates were harvested, fractionated on SDS-PAGE gels and probed for Nap1. Blots were reprobed for GAPDH to confirm sample loading. **a** Representative western blot and **b** summary of Nap1 levels detected after normalization for protein loading

Review of the RT-PCR data indicated that there were alterations in the processing of RNA encoding Nap1, a factor that interacts with both Tat and Rev and modulates their function [25, 26]. To assess whether our compounds were affecting Nap1 levels, western blots were performed. As shown in Fig. 11, 892 had no effect on Nap1 accumulation, while the effect of 833 was more variable. In contrast, 791 addition reduced expression of this factor by approximately 50%.

## Discussion

The central role that RNA processing plays in facilitating HIV-1 gene expression and replication makes it an attractive target for therapeutic intervention [1, 15]. Recent studies by several groups have established that small molecules (i.e. digoxin, chlorhexidine, 8-azaguanine, 5350150, IDC16, ABX464, 1C8) can be used to effectively alter viral RNA processing with limited or no effects on host cell viability [12–17]. Furthermore, the demonstration that ABX464 can suppress HIV-1 replication in humanized mice and has beneficial effects even after drug withdrawal indicates that such approaches might have benefits over currently approved therapeutics [16]. To expand the repertoire of small molecules affecting HIV-1 RNA processing, we have examined a subset of modulators of SMN2 splicing for their capacity to alter viral gene expression and identify three, 791, 833 and 892, as potent suppressors of HIV-1 protein expression (Gag, Env, Tat, and Rev) in the context of the HeLa rtTA HIV∆*Mls* cell line and 791 as an inhibitor of virus replication in PBMCs.

Given the activity of these compounds against HIV-1, we investigated whether there was any existing description of these compounds in scientific literature or patent applications that might provide insight into their mode of action. To date, no studies on either 791 or 833 have been published. However, there is limited information available for 892 as well as structures similar to 791 in other contexts. 892 and similarly structured compounds have been identified as putative activators of AMP-activated protein kinase (AMPK) (WO 2012027548), modulators of telomerase binding (WO 20122097600 and US 201200160260), and activators of histone deacetylase 1 (HDAC1) (WO 2010011318). Interestingly, a compound that is structurally similar to 892 was tested for inhibitory activity in the context of Hepatitis C virus (HCV) and shown to inhibit enzymatic activity of HCV protease by ~57% at 50 μM [27]. Two compounds resembling 791 were tested for inhibition of cyclin dependent kinase 2 (CDK2)/cyclin A. These compound differ in the side groups attached to the core pyrimidine ring structure. One compound, designated 12a, has a phenol group in place of the methyl group and a methyl group in place of the phenol ring with a chlorine in 791. 12a was shown to inhibit CDK2/cyclin A activity in vitro at an IC_50_ of 0.25 μM [28].

Despite the clear differences in structure between 791, 833 and 892, they have very similar effects on HIV-1 RNA processing and expression that are distinct from the activities described for other inhibitors of this stage of the virus lifecycle. Previously, we have demonstrated that digoxin, 8-azaguanine and 5350150 block HIV-1 Gag and Env expression and reduce accumulation of viral US and SS RNAs but do not share the same alterations in Tat and Rev [13, 14]. While digoxin reduced Rev accumulation, expression of the Rev-independent form of Tat (p16) was unaffected. In contrast, both 8-azaguanine and 5350150 had little effect on the levels of Tat or Rev protein but altered the localization of Rev within the cell. While studies on IDC16 and ABX464 have shown alterations in HIV-1 RNA processing and reduced virus replication, there is no report of their effects on expression of Tat or Rev [15, 16]. However, the effects of 791, 833, and 892 on viral RNA accumulation are comparable to the changes seen upon inhibition of Rev-induced transport by leptomycin B [29, 30] (Additional file 13: Figure S6), where treatment resulted in dramatic reduction of both US and SS RNA levels with little or no change in MS RNA abundance. Therefore, the loss of Rev function, either due to loss of the protein or Crm1 function, induces a shift in HIV-1 RNA accumulation due to either enhanced turnover of the US and SS RNAs in the nucleus or their splicing to generate MS RNA. Consequently, the effect of 791, 833, and 892 may not be attributable to direct effects on HIV-1 RNA processing but rather to changes in Rev protein synthesis/stability. As predicted from the loss of Rev, all three compounds induced nuclear retention of the remaining viral US RNA within the cell (Fig. 6). Consistent with action via reduction of Rev expression, compound addition induced only limited changes of the ~70 host RNA alternative splicing events initially tested (Fig. 9). Subsequent, more detailed analysis of 791 further validated these findings with only 15 of ~10,000 alternative splicing events showing changes of >10% in alternative exon inclusion (Fig. 10). 791 also had limited effects on overall gene expression, inducing changes of <fivefold in 85 genes and <tenfold of 4 genes of the ~10,000 genes detected in the assay (Fig. 10). In contrast, previous studies have demonstrated that T cell activation altered ~10% of >10,000 alternatively spliced events [31]. Although the compounds did not significantly affect cellular splicing events in general, the splicing of three exons, *fgfr1op2*, *macf1*, and *gm130/golga2*, was altered by all three compounds. Given that only a few cellular alternatively spliced events were appreciably changed among the total number of detected events, any changes that are common among the compounds would be predicted to be involved in their shared activity as inhibitors of HIV-1 gene expression.

The *macf1*, *gm130/golga2*, and *fgfr1op2* genes encode microtubule-actin crosslinking factor 1 (MACF1), Golgin A2, and fibroblast growth factor receptor 1 oncogene partner 2 (FGFR1OP2), respectively. MACF1 is a large protein that forms bridges between different cytoskeletal elements and has been shown to regulate microtubule dynamics by GSK3 signaling in skin stem cells and developing neurons [32, 33]. These studies found that GSK3 binds and phosphorylates MACF1, inhibiting MACF1’s ability to bind microtubules [32, 33]. Thus, MACF1 appears to be a downstream target of GSK3 signaling and further suggests that the compounds may impact the GSK3/Wnt signaling pathway. Similarly, Golgin A2 appears to be involved in cytoskeletal signaling pathways that regulate microtubule dynamics, as well as roles in the maintenance of the Golgi apparatus and secretory pathway [34]. Golgin A2 is phosphorylated by cyclin dependent kinase 1 (Cdk1)-cyclin B and cyclin dependent kinase 5 (Cdk5) [35, 36]. In turn, Golgin A2 binds and promotes the auto-phosphorylation of yeast Ste20-like kinases YST1 (human homologue is Stk25) and MST4, implicating the involvement of Golgin A2 in the MAPK signaling pathway [37]. In contrast to MACF1 and Golgin A2, the function of FGFR1OP2 is unknown, but is predicted to be translated into an evolutionarily conserved protein containing coiled-coil domains and may also play a role in related FGFR1 signaling pathways [38].

There was no overlap between the alternatively spliced and differentially expressed genes for 791 (Fig. 10d), consistent with mounting evidence from genome-wide studies in support of the understanding that most genes often undergo alternative splicing changes in protein isoforms without accompanying changes in overall transcript levels [31]. Only a few genes (84 for 791) were upregulated among the 11,406 genes examined. Of the genes that were differentially expressed, *trib3*, the gene encoding Tribbles pseudokinase 3 (TRIB3) was upregulated by over ninefold upon 791 treatment. TRIB3 is a putative protein kinase that is induced by transcription factor NFκB, and involved in numerous cellular processes [39]. Some of its roles include, inhibiting the activation of Akt, regulating activation of MAP kinases, and inhibiting APOBEC3A editing of nuclear DNA [39–41]. Since TRIB3 plays a role in regulating the PI3K/Akt signaling pathway and there is a dramatic difference in its expression with 791 treatment, it would be interesting to examine the involvement of TRIB3 during HIV-1 replication.

In the absence of significant changes in HIV-1 MS RNA abundance or shift in splice site usage upon addition of any of the compounds, the basis for the loss of Tat and Rev was not immediately evident. The limited effect on overall protein synthesis (as measured by SUnSET, see Fig. 7) indicates that the compounds are not general inhibitors of mRNA translation. The ability of the proteasome inhibitor MG132 to restore a significant amount of Tat accumulation indicates that Tat synthesis is occurring in the presence of the compounds. Tests to assess whether any of the compounds could directly affect Tat protein stability did not reveal any measurable changes (Additional file 6: Figure S4). However, these studies were performed by adding compound after translation was blocked by cycloheximide, so only existing Tat was measured. The failure to detect a decrease in p53 levels similar to those induced by curcumin, another modulator of Tat stability [24], suggested that these compounds are acting via different mechanisms. Our findings suggest that the compounds might be inducing a state within the cell that renders both Tat and Rev unstable and might require time for the transition to occur. Consistent with this hypothesis, we observed a reduction in Nap1 expression, a molecular chaperone and modulator of both Tat and Rev function [25, 26] upon addition of 791 (Fig. 11). Nap1 prevents the aggregation of Rev [25] and its overexpression increased Tat levels [26]. By binding Tat or Rev, Nap1 may reduce their availability for degradation by the proteasome.

In contrast to its effect on Tat, addition of MG132 did not restore but reduced Gag expression in the presence or absence of compound. Previously, Schubert et al. [42] demonstrated that MG132-induced proteasomal inhibition severely decreases the budding, maturation, and infectivity of HIV-1 by reducing the level of free ubiquitin in HIV-1-infected cells and thereby prevented mono-ubiquitination of p6^gag^ required for virus assembly and release. Thus, decreased p24 Gag levels with MG132 treatment is consistent with the requirement of functional proteasome for proteolytic processing of HIV-1 Gag [42].

Taken together, these results indicate that the compounds 791, 833, and 892 inhibit HIV-1 gene expression by inducing the loss of key early viral regulatory proteins, which, in turn, leads to a perturbation in the balance of HIV-1 RNAs and subsequent loss of viral structural proteins. While the detailed mechanism by which these compounds act remains to be determined, the description of their effects offer insights into new strategies to alter HIV-1 RNA processing. In addition to being structurally dissimilar to digoxin, 8-Azaguanine, and 5350150, these three compounds are also structurally distinct from NB-506, a splicing inhibitor that specifically blocks the kinase activity of DNA topoisomerase I [43], and ABX464 [16]. The fact that small molecular compounds with distinct structures can effect gene expression by modulating pre-mRNA splicing (NB-506, digoxin), mRNA transport (ABX464, 8-azaguanine, 5350150), and protein stability (791, 833, and 892) validates using small molecules as tools to probe components of RNA processing implicated in disease or viral infections. Furthermore, the similarities between the effects of these compounds and ABX464 on both HIV and cellular splicing events, suggest that more precise targeting of the affected processes could be used to inhibit HIV replication in vivo. There are many challenges in translating the effect of small molecules in vitro to their application as novel drugs in humans. The three compounds described here may not be directly applicable in patients, as their systemic effects and therapeutic dose ranges remain unknown. However, the ability of 791 to reduce HIV-1 replication in human PBMCs confirms activity in the natural context of HIV-1 infection. 791 inhibited HIV-1 BaL (R5-tropic) replication in peripheral blood mononuclear cells over 6 days post infection with >80% cell viability at concentrations up to 7.5 µM (Fig. 2). These results illustrate the promise of targeting HIV-1 RNA processing as a novel approach for the treatment of HIV-1 infection.

## Methods

### Indicator cell lines and viruses

Effects of compound treatment on HIV-1 gene expression were initially assessed in the context of HeLa rtTA HIV∆*Mls* cells stably transduced with an inducible Tet-On HIV-1 system (as previously described [12, 18, 19]). The provirus was modified by either deletion in the reverse transcriptase and integrase region of the pol gene by an *Mls*I restriction digest (HeLa rtTA HIV∆*Mls* cell line) or GFP fusion to Gag, deleting the PR and RT-coding regions (HeLa rtTA HIVGagGFP cell line). Tat and its TAR binding site are inactivated so that HIV-1 gene expression is only induced in the presence of doxycycline (dox). All cell lines were maintained in Iscove’s modified Delbecco’s medium (IMDM; Wisent) supplemented with 10% (vol/vol) fetal bovine serum (FBS, Wisent), 1% penicillin/streptomycin (P/S, Wisent) and 0.2% Amphotericin B (Wisent). Indicator cell line CEM-GXR was obtained from Dr. Mark Brockman (Simon Fraser University). The RTI-resistant virus (E00443) was obtained from Dr. Zabrina Brumme (Simon Fraser University). The HIV-1 clade A (97USSN54 (N54)), HIV-1 clade B (IIIB), integrase inhibitor resistant virus (11845), protease inhibitor resistant virus (2948) were obtained through the NIH AIDS Research and Reference reagent program, Division of AIDS, NIAID, NIH).

### Compound treatment assay

The compounds used in the treatment assay were obtained from ChemBridge. All compounds were solubilized to 10 mM or 1 mM stock concentrations in dimethyl sulfoxide (DMSO) and stored at −20 °C for subsequent experiments. Cells were incubated for 3–5 h in the presence of the compounds prior to induction with doxycycline (Dox) at a final concentration of 2 μg/mL. 24 h post compound treatment, culture medium was harvested, adjusted to 1% Triton X-100, and incubated at 37 °C for 1 h for p24 antigen ELISA. Cells were harvested in 2 mM EDTA, 1xPBS. RNA was isolated using Aurum Total RNA extraction kits (Bio-Rad), while total protein was extracted with RIPA buffer (1% NP-40, 0.1% SDS, 0.5% sodium deoxycholate, 150 mM NaCl, 50 mM Tris–HCl).

To examine the effect of compounds on viral replication, studies were carried out in peripheral blood mononuclear cells (PBMCs). PBMCs were isolated from healthy (HIV-uninfected) volunteer blood donors as described by Dobson-Belaire et al. [44]. Informed consent was obtained from participants in accordance with the guidelines for conduct of clinical research at the University of Toronto and St. Michael’s Hospital, Toronto, Ontario, Canada. Stored PBMCs were thawed, washed with RPMI 1640 complete medium and cultured in RPMI 1640 complete medium containing 2 μg/mL of PHA-L (Sigma-Aldrich) and 20 U/mL of IL-2 (BD Pharmingen) for 72 h. Subsequently, cells were counted and a portion of the cells was separated to another tube for uninfected control treatments. The remaining PBMCs were resuspended in media containing HIV-1 BaL at a multiplicity of infection (MOI) of approximately 0.01 and infected by spinoculation, following which cells were washed twice with room temperature RPMI 1640 complete medium and resuspended to a concentration of 5 × 10^5^ cells/mL in complete RPMI 1640 containing 20 U/mL of IL-2. Compounds were added to infected PBMCs or uninfected control PBMCs. Azidothymidine (AZT, Sigma-Aldrich) was used as control treatment at a final concentration of 3.74 μM. On day 4 post infection, culture medium was replenished with the compounds and IL-2 in fresh complete RPMI 1640. On days 2, 4, 6 and 8 post infection, culture supernatant was harvested, virus lysed by adjusting to 1% TritonX-100 and stored at −20 °C for p24 antigen ELISA. A fraction of the culture was harvested to assess percent cell viability by trypan blue exclusion using Glasstic slides (Kova). Relative percent cell viability in compound treated samples versus DMSO-control treated samples was calculated as follows: (total viable cells/total cells)_compound_/(total viable cells/total cells)_DMSO_.

To assess the activity of compounds against HIV-1 strains having resistance to various ART drugs, assays were performed in the context of CEM-GXR cells. These cells express CD4, CXCR4, and coreceptor, CCR5 as well as an exogenous Tat-driven LTR-GFP expression cassette. Cultures were infected with different HIV-1 strains of interest, differing on their subtypes (A and B), and resistance to three major drug targets (reverse transcriptase, protease, and integrase). The culture contains serial dilutions of the molecule in the final concentrations from 0.156 to 5 μM for 833; 725 nM to 46.87 μM for 791; 1.95 to 62.5 μM for 892. Antiviral activity was evaluated in the assay by measuring inhibition of HIV-1 spread in a coculture of CEM-GXR cells containing 1% of HIV-1 infected (GFP positive) cells using flow cytometric analysis (GuavaSoft 2.2 software, Guava HT8, Millipore). To estimate the viable cell counts, the gate in a flow cytometer (Guava HT8) was set to cover 95% of the freshly passaged uninfected CEM-GXR cell or using ViaCount™. The same parameter was employed to gate viable cells in the inhibition assay and the number of gated cells was obtained by GuavaSoft 2.2 software. For assays using ViaCount™, sample acquisition and data analysis were performed with the selection of EasyFit analysis feature using the ViaCount™ software module.

### Analysis of protein expression

ELISA for p24 Gag antigen was performed on cell supernatants using kits purchased from Frederick National Laboratory for Cancer Research (Leidos) or XpressBio extended range kit and performed according to manufacturer’s instructions. Protein concentration in cell lysates was quantified by Bradford assay and equal amounts of protein run on 7, 10, 12, or 14% SDS-PAGE, depending on the protein of interest. Following transfer to PVDF (BioRad or Perkin-Elmer), blots were blocked in either 5% Milk-PBS-T (5% Milk, 0.05% Tween-20, 1× PBS) or 3% BSA-PBS-T (3% BSA, 0.05% Tween-20, 1× PBS) prior to incubating with primary antibody (all diluted in 3% BSA-PBS-T). Primary antibodies used were: purified mouse anti-p24 supernatant from anti-HIV-1 Gag hybridoma 183, mouse anti-gp120 hybridoma 902 (NIH AIDS Reagent Program), mouse monoclonal antibody to HIV-1 Rev (Abcam), rabbit polyclonal antibody to HIV-1 Tat (Abcam), mouse monoclonal antibody to p53 (Santa Cruz), rabbit purified IgG for Nap1 (provided by L. Frappier), rabbit polyclonal antibody to GAPDH (Sigma-Aldrich), or mouse monoclonal antibody to α-Tubulin (Sigma-Aldrich). Following incubation with appropriate secondary antibody, blots were visualized by ECL, ECL Plus (Perkin-Elmer), or Clarity Western ECL substrate (BioRad). Quantification of the relative intensity of the detected bands was done using ImageLab software and normalized to corresponding bands of the loading control (GAPDH or α-Tubulin).

### Compound toxicity assays

Effects of compound treatment on cellular metabolism was assessed by an XTT-based in vitro toxicology assay kit (Sigma-Aldrich) or Trypan blue exclusion (Life Technologies) as proxy for degree of cytotoxicity and expressed relative to DMSO control treatment. For measurement of cell viability using XTT, culture media was removed after 24, 72, and 96 h of compound treatment, replaced with 20% XTT solution and incubated at 37 °C in a 5% CO_2_ humidified incubator for 2–6 h, and relative cell viability was measured in compound treated cells relative to DMSO-treated cells. Cell viability measurements in CEM-GXR cells are described above.

### RNA analysis

Samples were processed and assayed as previously described using the BioRad Aurum Total RNA Lysis Kit (BioRad) as per manufacturer’s instructions with the addition of Turbo DNase (Ambion). Purified RNA (0.5–2 μg) was reverse transcribed using M-MLV (Invitrogen) to generate complementary DNA (cDNA). HIV-1 and actin mRNA levels in DMSO- and compound-treated samples were quantified by qPCR using the Mastercycler ep realplex (Eppendorf) as described by Wong et al. [12]. Gene quantification was evaluated using the absolute quantification method, normalized to β-actin expression, and expressed relative to DMSO-treatment.

The effect of compound treatment on splice site selection within the HIV-1 MS RNA class was analyzed by radioactive RT-PCR as described previously [20]. Radioactive reaction products were resolved on 6% denaturing polyacrylamide gels (8 M Urea, 1xTBE) and detection using a Typhoon 9400 PhosphorImager (Amersham). Gel densitometry was performed using ImageJ software (NIH) to calculate mRNA levels of HIV-1 MS mRNA isoforms, measured as the density of an individual isoform divided by the total density of all visible viral RNA species in a sample.

Changes in HIV-1 US RNA subcellular distribution in response to compound treatment was analyzed by fluorescent in situ hybridization in HeLa HIVrtTA GagGFP cells, as described by Wong et al. [14]. Following washing to remove unbound probe, nuclei were stained with DAPI and images were acquired using a Leica DMR microscope at 630× magnification.

To assess the effect of compounds on expression/alternative splicing of cellular RNA, two approaches were used. For both, total RNA was prepared from DMSO or compound-treated cells. In the first assay, samples were assayed by medium throughput RT-PCR to determine the inclusion levels of alternatively spliced exons and splice sites located in 73 selected events. 73 primer sets containing a fluorescently (5-FAM) labeled primer for each, were used in RT-PCR. PCR products generated were denatured in formamide and quantified using ABI Prism capillary sequencer (Life Technologies). The fragment analysis was performed on the PeakScanner software (Life Technologies) in batch mode and automated using custom scripts written in Python. The inclusion level of each exon was calculated as the amount of transcripts carrying the alternative exon relative to the total amount of all transcripts detected in the PCR reaction and results are summarized for compound-treatment in comparison to DMSO treatment. In the second assay, extracted RNA was processed using the Illumina TruSeq RNA Sample Preparation Kit (Illumina) according to the manufacturer’s instructions. The cDNA library generated was validated (passed quality control on a Bioanalyzer 1000 DNA chip (Agilent)), normalized and pooled for cluster generation. cDNA libraries were sequenced on the Illumina HiSeq 2500 (paired-end, 125 bp) with version 4 chemistry following manufacturer’s protocols. The full human genome and transcriptomic sequences were downloaded from the UCSC Genome Browser (genome.ucsc.edu) database and Ensembl (www.ensembl.org), respectively, as described by Irimia et al. [45]. Exon annotations and genomic coordinates for alternative splicing (AS) analysis were derived from tables downloaded from the UCSC Genome Browser database. To determine gene expression (GE) or alternative splicing (AS) changes in an unbiased way, the effective number of unique mappable positions in each transcript (i.e. the effective length) was determined by aligning sequences with unique transcriptomic alignment to the human genome using Bowtie [46]. Reads with one unique genomic alignment were then aligned against the canonical transcriptome and, for each transcript, the number of reads with one unique transcriptomic alignment were counted. The expression level of genes was quantified as corrected ‘reads per kilobase of exon model per million mapped reads’ (cRPKM), a widely used metric to estimate gene expression levels. The expression cutoff was 0.5 cRPKM, corresponding to the transcript of the gene being present if there were ≥10 reads that mapped uniquely to a single genomic locus. Approximately 19,847 Ensembl annotated protein-coding genes were compared to create a gene list of differentially expressed genes. Genes were considered differentially expressed if fold changes in cRPKM was ≥2 in compound-treated versus DMSO-treated samples.

To obtain the percent spliced in (PSI) estimation, the following procedure was followed. Every internal exon in each annotated transcript was considered a potential “cassette” exon as described previously [45]. Briefly, each “cassette” AS event was defined by three exons: C1, A and C2, where A was the alternative exon, and C1 and C2 were the 5′ and 3′ constitutive exons, respectively. For each event, spliced junctions were defined as follows: C1A (connecting exons C1 and A), AC2 (connecting exons A and C2), and one alternative junction, C1C2 (connecting exons C1 and C2). For each sample, the corresponding mRNA-Seq data were aligned against the human genome using Bowtie, allowing for a maximum of two mismatches. Reads that did not map to the genome were then aligned to the full non-redundant set of junction sequences and, for each junction, the number of reads with one unique alignment mapping to it were counted. For each junction, the corresponding read count was normalized for its mapping ability by multiplying the read count by the ratio between the maximum number of mappable positions and its effective number of unique mappable positions (as defined above). The percent inclusion, or “percent spliced-in” (PSI) value, for each internal exon was defined as: PSI = 100× average (#C1A, #AC2)/(#C1C2 + average(#C1A, #AC2)), where #C1A, #AC2 and #C1C2 were the normalized read counts for the associated junctions. Exons were considered alternative in a sample if 5 ≤ PSI ≤ 95. In addition “high confidence” PSI levels were defined as those PSI values that fulfilled the following specific coverage and balance criteria: max(min(#C1A, #AC2), #C1C2) ≥5 AND min(#C1A, #AC2) + #C1C2 ≥10 and |log2(#C1A/#AC2)| ≤1 OR max(#C1A, #AC2) <#C1C2. The goal of the first criterion was to ensure enough read coverage for sufficient precision and resolution in the estimation of PSI levels. The goal of the second criterion was to exclude AS events where there was a high imbalance in read counts between the two junctions formed by exon inclusion since these imbalances can confound PSI estimates for cassette AS events. For comparison of AS levels between pairs of samples, Pearson correlation was applied to PSI levels. Events were considered differentially spliced between DMSO- and compound-treated samples if changes in PSI levels were ≥10.

### Monitoring protein synthesis by SUnSET

The effect of the compounds on nascent protein synthesis was measured by surface sensing of translation (SUnSET) as described by Schmidt et al. [23]. Cells were incubated with puromycin, an aminoacyl tRNA analog, to allow puromycin incorporation into newly translated peptides and prevention of further ribosomal elongation by chain termination. To assess the effect of the compounds on protein translation, cells were prepared and treated as described above, but were incubated with 10 μg/mL of puromycin for a period of 30 min prior to harvesting cell lysates for protein analysis. Protein concentration of cell lysates was quantified by Bradford assay and equal amounts of protein (30–35 μg) was run on either 10% or 4–15% (gradient) gels. Following transfer to PVDF (BioRad), blots were probed with mouse monoclonal antibody to puromycin (anti-12D10, EMD Millipore). Blots were developed using ECL Plus (Perkin-Elmer) or Clarity (BioRad) and imaged using the ChemiDoc MP Imager (BioRad). To quantify the levels of protein synthesis, the volume intensity in each lane of compound-treated sample was calculated relative to the DMSO-treated sample and normalized to GAPDH loading control using ImageLab software (BioRad) from at least four independent experiments.

### Effect of compounds on HIV-1 Tat stability

To assess effect of compounds on HIV-1 Tat stability, the decay of HIV-1 Tat levels was compared between DMSO-treated and compound-treated protein lysates in the presence of cycloheximide. In the first set of experiments, HIV-1 gene expression was induced with doxycylin (dox) for 24 h then 10 μg/mL cycloheximide was added to block new protein synthesis in combination with either DMSO or the compounds. Cells were harvested every 2 h and Tat protein levels measured by western blot. Quantification of the relative intensity of the detected bands was performed using ImageLab software (BioRad) and normalized to corresponding bands of the loading control (GAPDH) from at least three independent experiments. To determine whether the compounds’ effect on HIV-1 gene expression could be reversed by inhibition of the proteasome, compound treatment assay was performed as previously described with the addition of 10 μM MG132 (Sigma-Aldrich) to compound-treated cells 8 h prior to harvesting. Equal amounts of protein were run on 13 or 14% gels by SDS-PAGE, blotted and probed with antibodies for Tat and GAPDH. Quantification of the relative intensity of the detected bands was performed using ImageLab software (BioRad) and normalized to corresponding bands of the loading control (GAPDH) from at least three independent experiments.

### Statistical analysis

In vitro experiments were all performed on at least three separate occasions and are represented as the mean ± the standard error (SEM) of the experiment, unless otherwise stated. Statistical significance comparisons between two samples were calculated using the paired two-tailed student’s t test (Microsoft Excel) and graphs were generated using Prism 5.0 software (GraphPad). Significant differences are represented by comparison to DMSO-treated control samples with the following legend: *p ≤ 0.05, **p ≤ 0.01 and ***p ≤ 0.001. Significance levels of *p* ≤ 0.05 were considered statistically significant.

## Additional files

**Additional file 1: Figure S1.** 791 and 833 Suppress HIV-1 Gene Expression in SupT1 24NESLG Cell Line. **a** Schematic of HIV-1 provirus used to generated the stably transduced SupT1 cell line [47]. **b** Effect of 791 and 833 on Gag expression in SupT1 NLESG cells. Cells were treated with indicated concentrations of 791/833 then HIV-1 expression induced by PMA addition. Media harvested after 24 h was subsequently assayed using Gag (p24) ELISA. Cell viability was measured by an XTT assay. 892 was not active in this cell line at the concentrations tested

**Additional file 2: Figure S7.** Effect of compounds on CEM-GXR cell viability. Evaluation of 791, 833, and 892 on CEM-GXR cell viability in the Guava ViaCount assay. Cells were analyzed after 24 h incubation with the compounds at the indicated concentrations ranging between 0–47 μM (791), 0–5 μM (833), or 0–62.5 μM (892). Results are expressed as the percentage (%) of viable cells relative to DMSO treatment ±SEM of two independent experiments performed in triplicate. Cytotoxic concentration resulting in the death of 50% of the host cells (CC50) relative to DMSO measured by ViaCount assay (Millipore) are listed in Table 1

**Additional file 3: Table S1.** Mutations in HIV-1 proteins associated with resistance to ARVs

**Additional file 4: Figure S2.**Pattern of HIV-1 RNA splicing. Shown at the top is the organization of the HIV-1 proviral genome indicating the position of the multiple 5′ splice donor sites (SD1–SD4) and 3′ splice acceptor sites (SA1–SA7) used in splicing of pre-mRNA. In the middle is an illustration of the alternatively spliced RNAs generated by processing of the HIV-1 genomic RNA. Indicated are the common (open boxes) and alternative exons (closed boxes) used in the generation of the SS (4 kb) and MS (1.8 kb) viral RNAs. At the bottom is a list of the nomenclature used to refer to the exon composition of the individual RNAs generated for both the SS and MS classes of HIV-1 RNAs

**Additional file 5: Figure S3.** Characterization of HeLa rtTA HIVGagGFP cell line. **a** Representative images of HeLa rtTA HIVGagGFP C7 cells treated with DMSO in the absence (uninduced) or presence (induced) of doxycyclin (N ≥ 3). Cells were viewed at 630X (oil immersion) magnification. Images are cropped to show a representative field of view. **b** The dose range of the compounds which inhibit HIV-1 GagGFP expression in HeLa rtTA HIVGagGFP C7 cells was measured by mean fluorescence intensity and expressed relative to fluorescence intensity in DMSO-treated samples (N ≥ 3, *p ≤ 0.05, **p ≤ 0.01, and ***p ≤ 0.001). The effect of the compounds on cellular metabolism at the indicated concentrations was measured using an XTT assay as a readout of viable cells and expressed relative to absorbance reads of DMSO-treated samples (N ≥ 3, *p ≤ 0.05, **p ≤ 0.01, and ***p ≤ 0.001). Error bars indicate standard error of the mean (SEM)

**Additional file 6: Figure S4.** 791, 833 or 892 do not alter the half-life of HIV-1 Tat relative to DMSO. **a** Representative blots showing the decay of Tat protein in the presence of cycloheximide (10 µg/ml) and DMSO or indicated compounds (N ≥ 3, except for 833, N = 1–2). MG132 (10 μM) was added for 8 h as an additional control to determine whether inhibition of the proteasome prevents protein degradation. All uninduced (unind.) and 0 h samples were treated with DMSO. GAPDH serves as loading control. **b** Summary of effect of compounds on HIV-1 Tat degradation. Band volume intensities of both p14 and p16 Tat isoforms were calculated for each treatment relative to that of the DMSO control treatment and were then normalized to corresponding GAPDH bands (N ≥ 3, except for 833, N = 1–2). Error bars depict standard error of the mean, if possible

**Additional file 7: Table S2.**Dataset from RT-PCR determination of the alternative splicing changes in cells treated with 9147791

**Additional file 8: Table S3.**Dataset from RT-PCR determination of the alternative splicing changes in cells treated with 5227833

**Additional file 9: Table S4.** Dataset from RT-PCR determination of the alternative splicing changes in cells treated with 5193892

**Additional file 10: Table S5.** Dataset from RNA-seq showing genes with alternative splicing changes in cells treated with 9147791

**Additional file 11: Table S6.**Dataset from RNA-seq quantitating the expression levels of genes in cells treated with 9147791

**Additional file 12: Figure S5.**791 has reduced toxicity in comparison to 833 and 892. The graph shows cell proliferation as measured by XTT assay 1, 3, and 4 days post-treatment with the compounds relative to DMSO-treated HeLa rtTA HIV∆*Mls* cells (N = 3). Error bars depict standard error of the mean and *, **, and *** indicate P values ≤ 0.05, 0.01, and 0.001, respectively

**Additional file 13: Figure S6.** Leptomycin B treatment reduces HIV-1 US and SS RNA Accumulation. 293T cells were transfected with pHxbc2 R-/RI-, an HIV-1 proviral clone which does not express either reverse transcriptase or integrase. Following overnight incubation with transfection cocktail, cells were washed and incubated in media ±20 nM leptomycin B (+LB) for 24 h. Cells were subsequently harvested and total RNA extracted. Levels of HIV-1 US, SS and MS RNAs was determined by qRTPCR as outlined. Shown is the result of N = 3 independent assays
