# Supplementary figures and images for: Identification of small molecule modulators of HIV-1 Tat and Rev protein accumulation
Source: Retrovirology. 2017 Jan 26;14:7. doi: 10.1186/s12977-017-0330-0 (PMC5267425; doi:10.1186/s12977-017-0330-0)

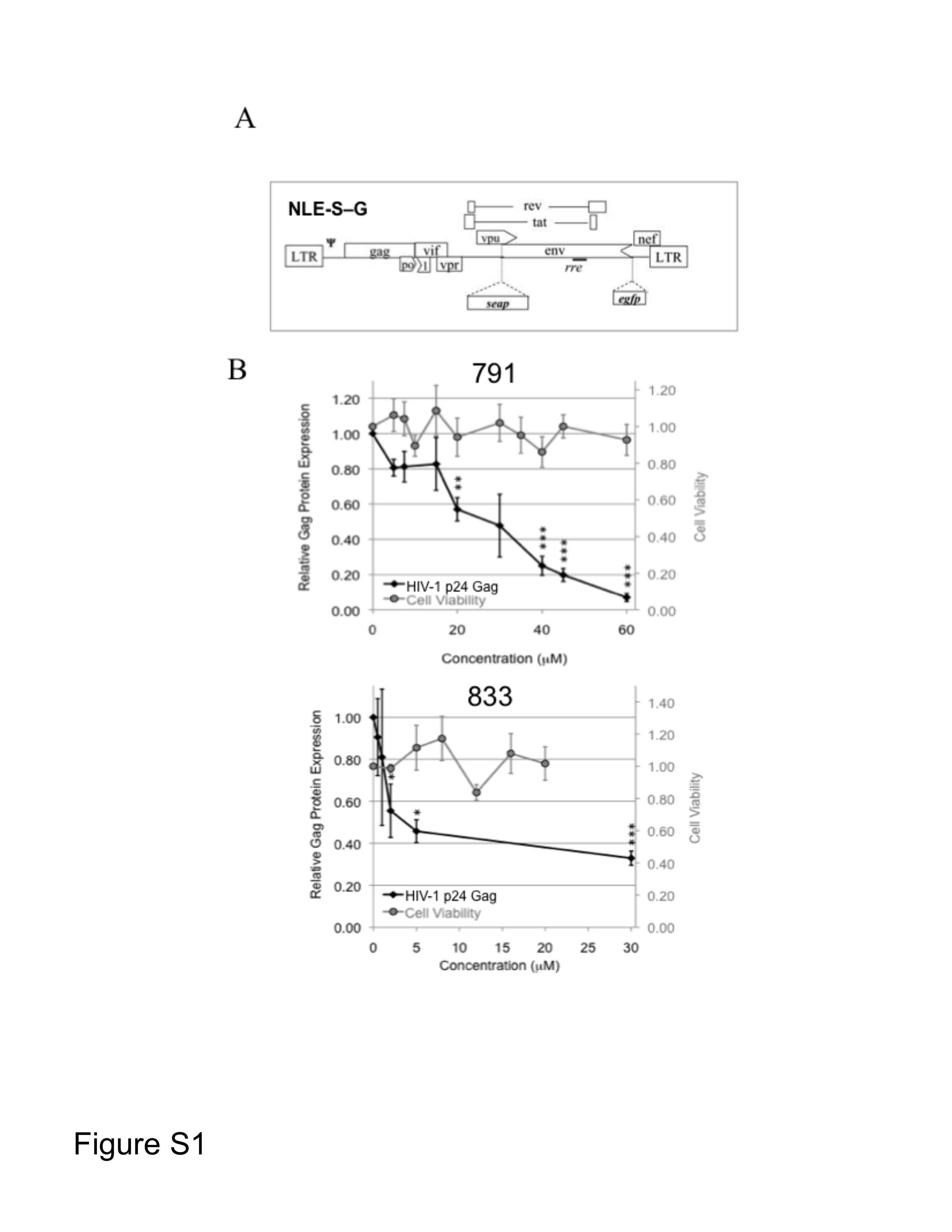

Supplement: Supplementary file 1 — Additional file 1: Figure S1. 791 and 833 Suppress HIV-1 Gene Expression in SupT1 24NESLG Cell Line. a Schematic of HIV-1 provirus used to generated the stably transduced SupT1 cell line [47]. b Effect of 791 and 833 on Gag expression in SupT1 NLESG cells. Cells were treated with indicated concentrations of 791/833 then HIV-1 expression induced by PMA addition. Media harvested after 24 h was subsequently assayed using Gag (p24) ELISA. Cell viability was measured by an XTT assay. 892 was not active in this cell line at the concentrations tested [file 12977_2017_330_MOESM1_ESM.tif]

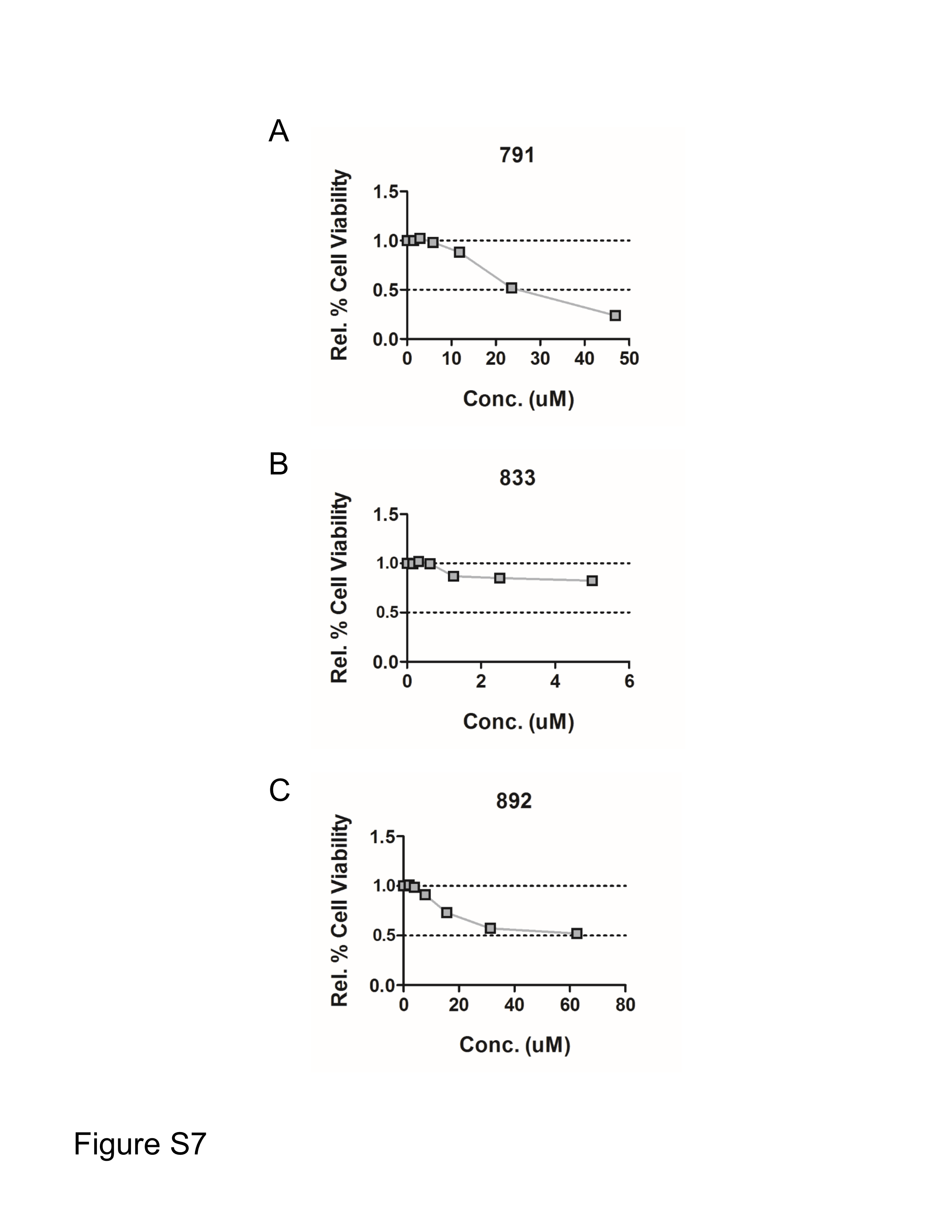

Supplement: Supplementary file 2 — Additional file 2: Figure S7. Effect of compounds on CEM-GXR cell viability. Evaluation of 791, 833, and 892 on CEM-GXR cell viability in the Guava ViaCount assay. Cells were analyzed after 24 h incubation with the compounds at the indicated concentrations ranging between 0–47 μM (791), 0–5 μM (833), or 0–62.5 μM (892). Results are expressed as the percentage (%) of viable cells relative to DMSO treatment ±SEM of two independent experiments performed in triplicate. Cytotoxic concentration resulting in the death of 50% of the host cells (CC50) relative to DMSO measured by ViaCount assay (Millipore) are listed in Table 1 [file 12977_2017_330_MOESM2_ESM.tif]

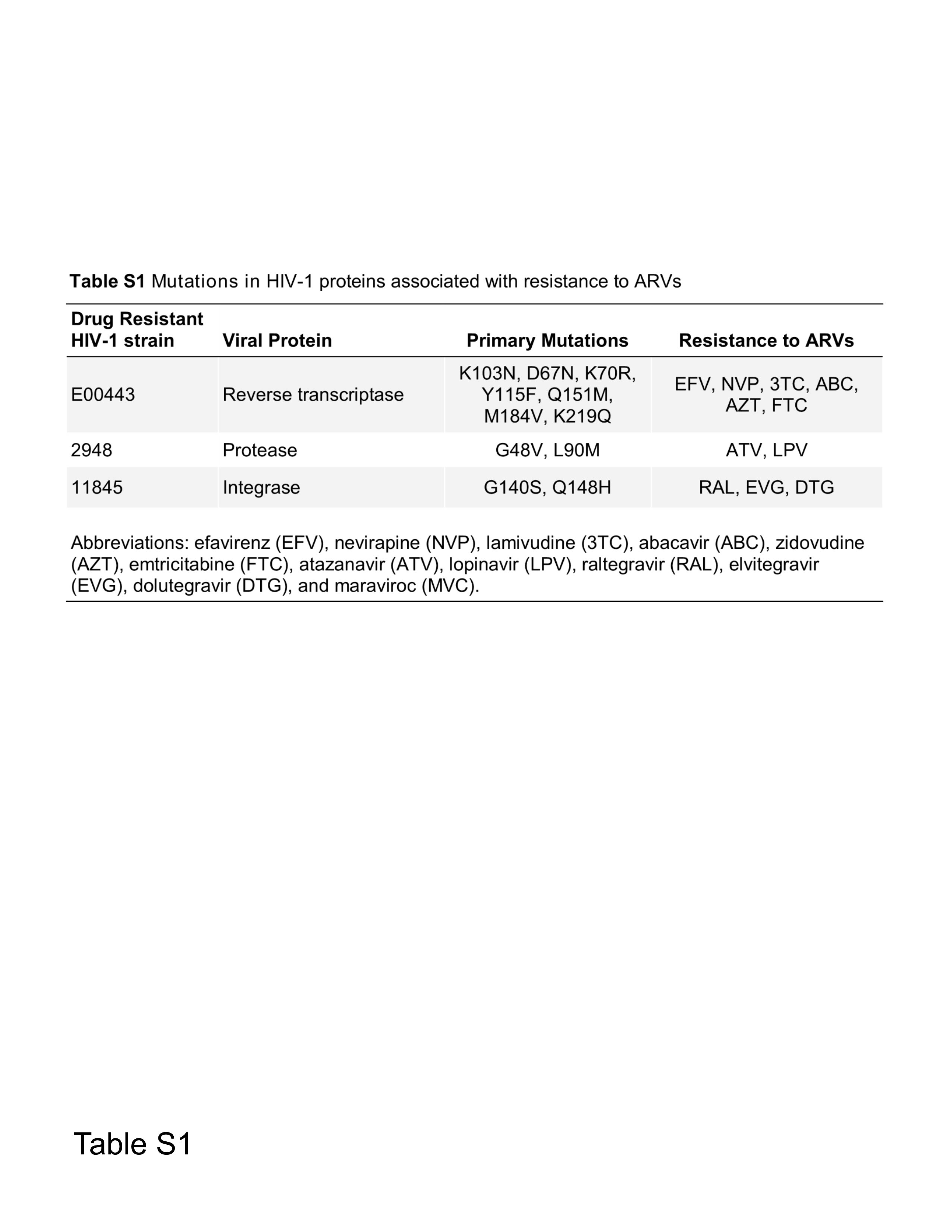

Supplement: Supplementary file 3 — Additional file 3: Table S1. Mutations in HIV-1 proteins associated with resistance to ARVs [file 12977_2017_330_MOESM3_ESM.tif]

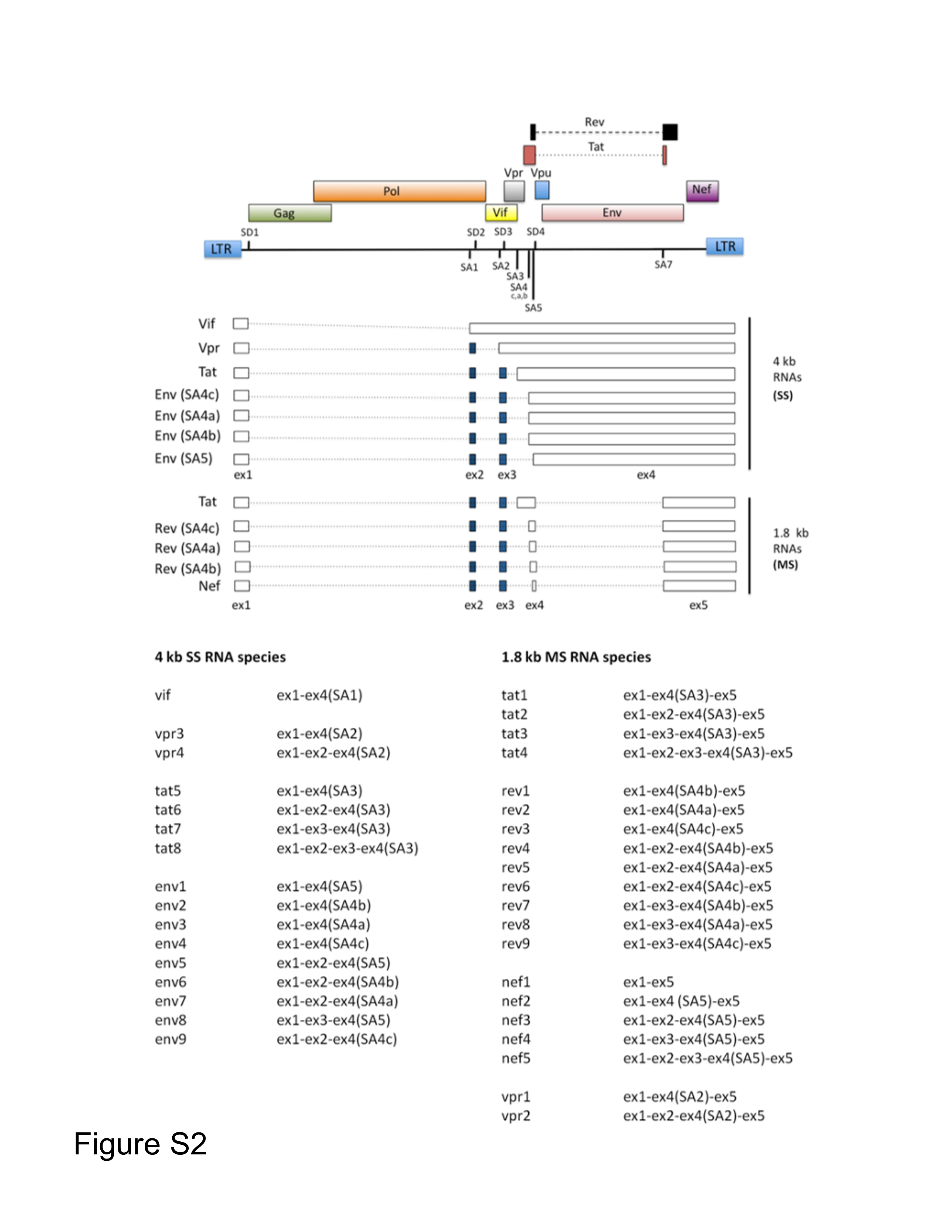

Supplement: Supplementary file 4 — Additional file 4: Figure S2.Pattern of HIV-1 RNA splicing. Shown at the top is the organization of the HIV-1 proviral genome indicating the position of the multiple 5′ splice donor sites (SD1–SD4) and 3′ splice acceptor sites (SA1–SA7) used in splicing of pre-mRNA. In the middle is an illustration of the alternatively spliced RNAs generated by processing of the HIV-1 genomic RNA. Indicated are the common (open boxes) and alternative exons (closed boxes) used in the generation of the SS (4 kb) and MS (1.8 kb) viral RNAs. At the bottom is a list of the nomenclature used to refer to the exon composition of the individual RNAs generated for both the SS and MS classes of HIV-1 RNAs [file 12977_2017_330_MOESM4_ESM.tif]

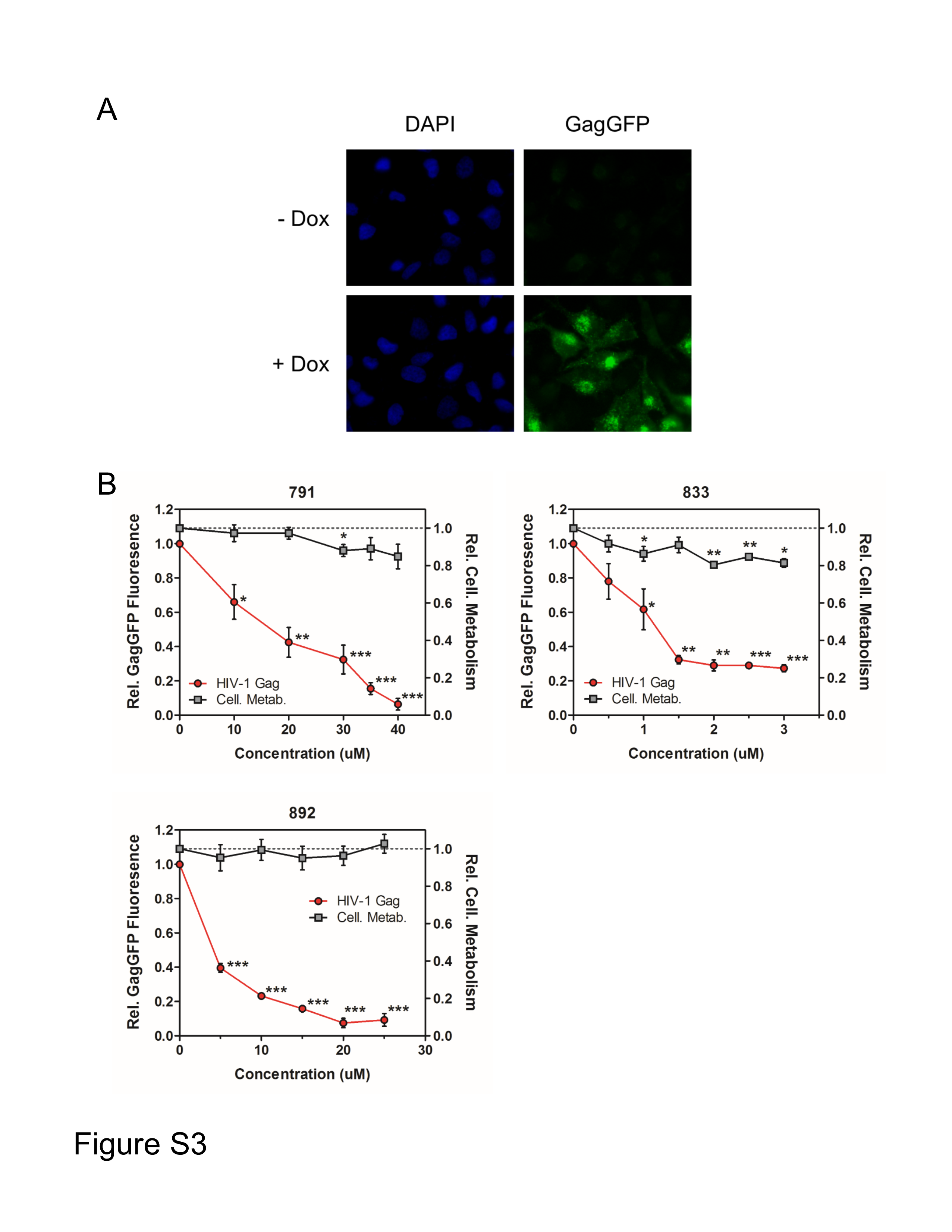

Supplement: Supplementary file 5 — Additional file 5: Figure S3. Characterization of HeLa rtTA HIVGagGFP cell line. a Representative images of HeLa rtTA HIVGagGFP C7 cells treated with DMSO in the absence (uninduced) or presence (induced) of doxycyclin (N ≥ 3). Cells were viewed at 630X (oil immersion) magnification. Images are cropped to show a representative field of view. b The dose range of the compounds which inhibit HIV-1 GagGFP expression in HeLa rtTA HIVGagGFP C7 cells was measured by mean fluorescence intensity and expressed relative to fluorescence intensity in DMSO-treated samples (N ≥ 3, *p ≤ 0.05, **p ≤ 0.01, and ***p ≤ 0.001). The effect of the compounds on cellular metabolism at the indicated concentrations was measured using an XTT assay as a readout of viable cells and expressed relative to absorbance reads of DMSO-treated samples (N ≥ 3, *p ≤ 0.05, **p ≤ 0.01, and ***p ≤ 0.001). Error bars indicate standard error of the mean (SEM) [file 12977_2017_330_MOESM5_ESM.tif]

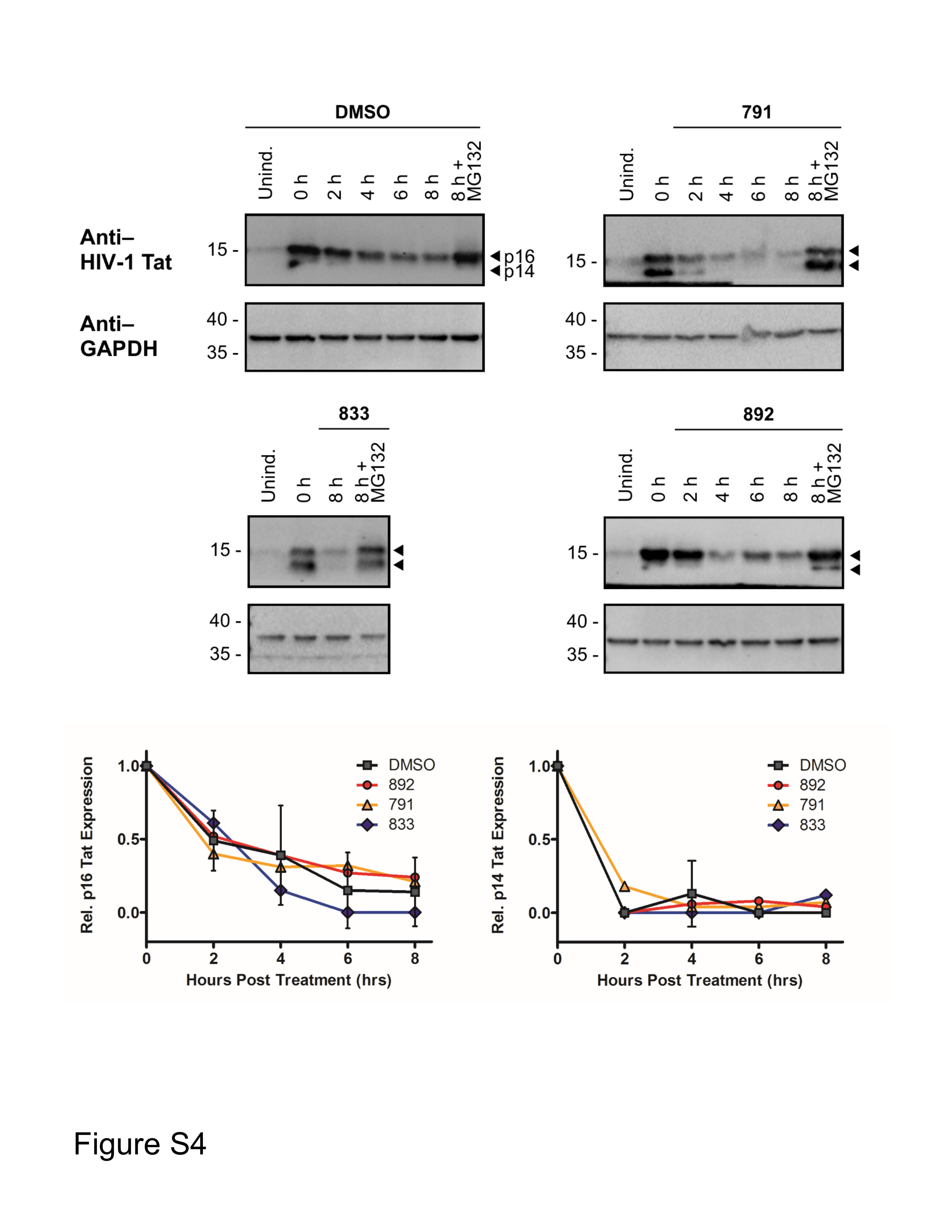

Supplement: Supplementary file 6 — Additional file 6: Figure S4. 791, 833 or 892 do not alter the half-life of HIV-1 Tat relative to DMSO. a Representative blots showing the decay of Tat protein in the presence of cycloheximide (10 µg/ml) and DMSO or indicated compounds (N ≥ 3, except for 833, N = 1–2). MG132 (10 μM) was added for 8 h as an additional control to determine whether inhibition of the proteasome prevents protein degradation. All uninduced (unind.) and 0 h samples were treated with DMSO. GAPDH serves as loading control. b Summary of effect of compounds on HIV-1 Tat degradation. Band volume intensities of both p14 and p16 Tat isoforms were calculated for each treatment relative to that of the DMSO control treatment and were then normalized to corresponding GAPDH bands (N ≥ 3, except for 833, N = 1–2). Error bars depict standard error of the mean, if possible [file 12977_2017_330_MOESM6_ESM.tif]

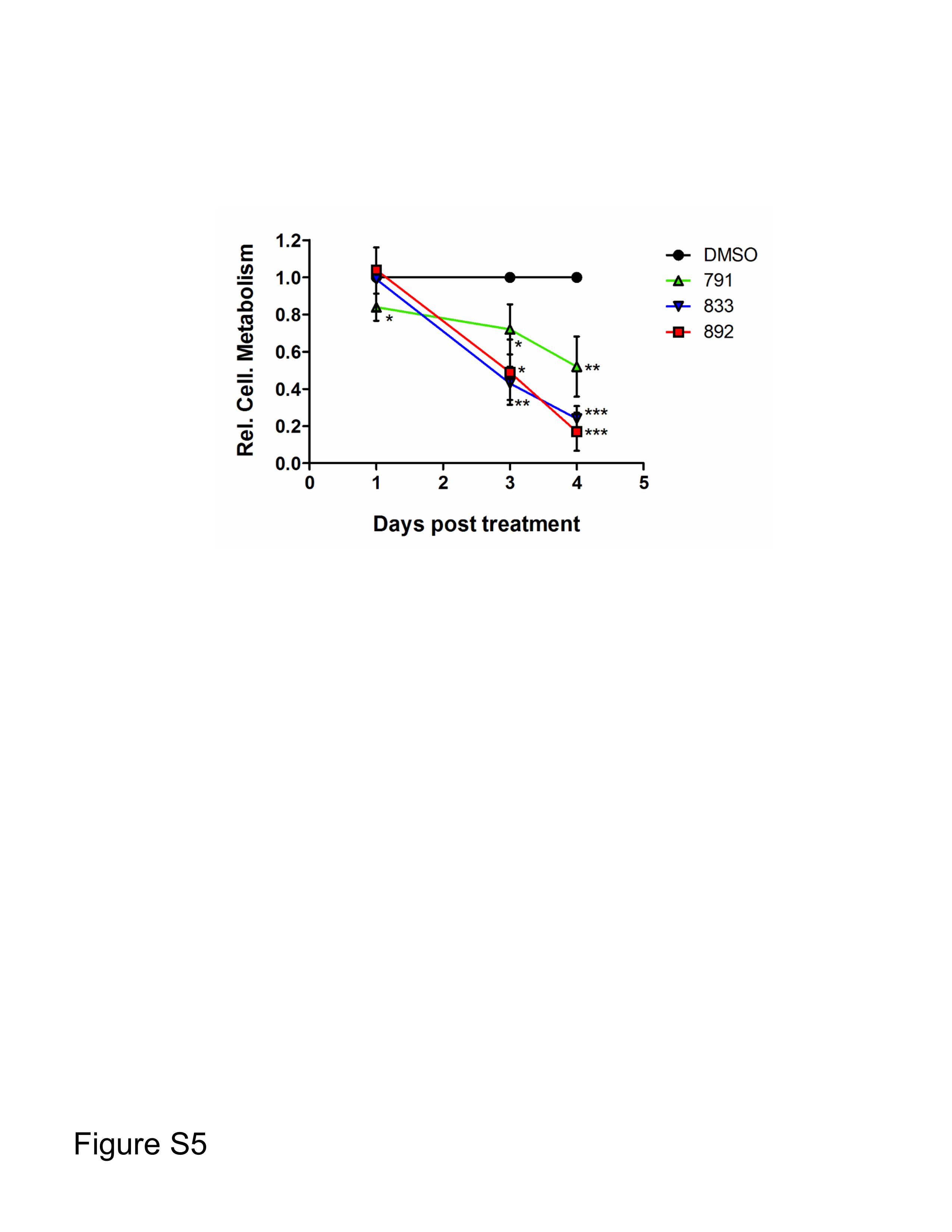

Supplement: Supplementary file 12 — Additional file 12: Figure S5.791 has reduced toxicity in comparison to 833 and 892. The graph shows cell proliferation as measured by XTT assay 1, 3, and 4 days post-treatment with the compounds relative to DMSO-treated HeLa rtTA HIV∆Mls cells (N = 3). Error bars depict standard error of the mean and *, **, and *** indicate P values ≤ 0.05, 0.01, and 0.001, respectively [file 12977_2017_330_MOESM12_ESM.tif]

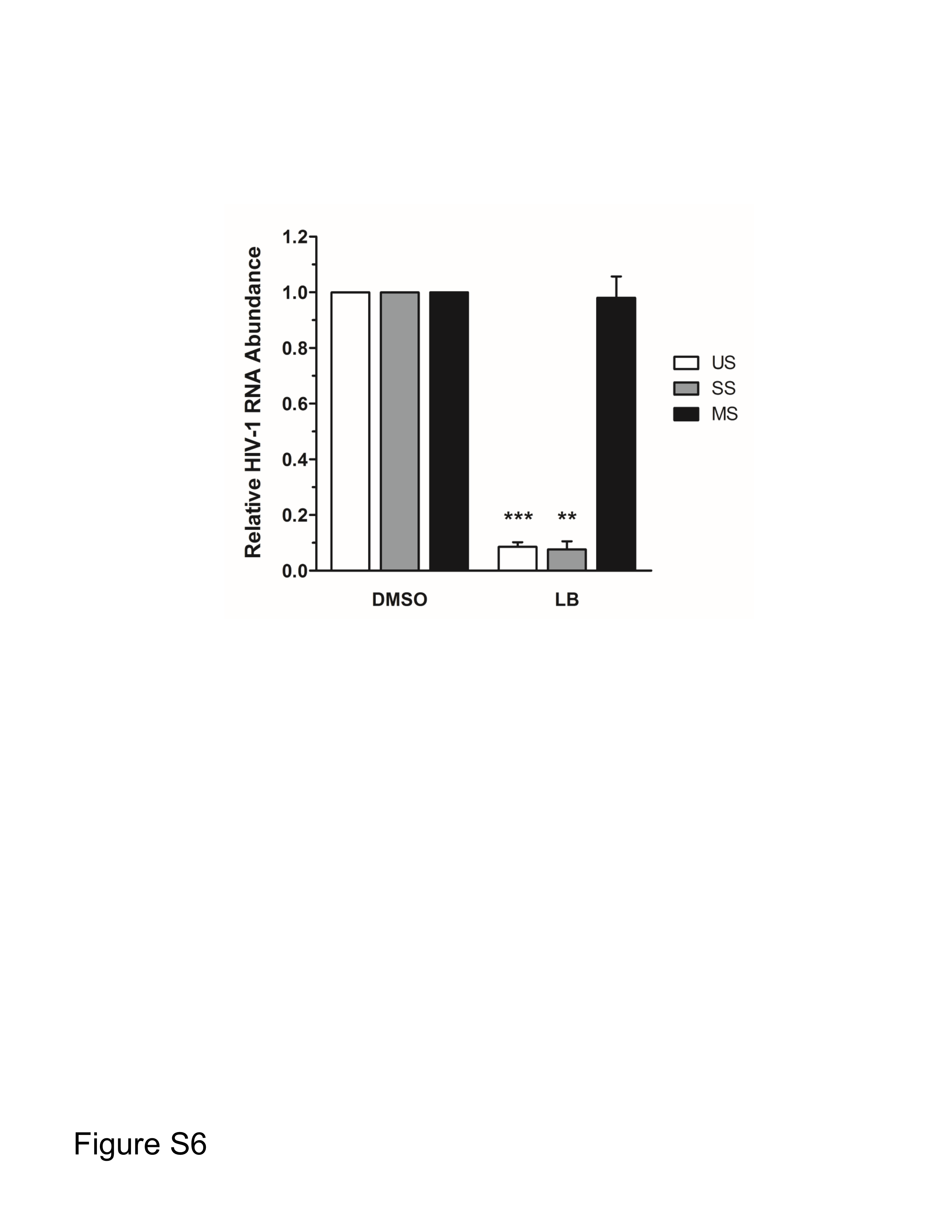

Supplement: Supplementary file 13 — Additional file 13: Figure S6. Leptomycin B treatment reduces HIV-1 US and SS RNA Accumulation. 293T cells were transfected with pHxbc2 R-/RI-, an HIV-1 proviral clone which does not express either reverse transcriptase or integrase. Following overnight incubation with transfection cocktail, cells were washed and incubated in media ±20 nM leptomycin B (+LB) for 24 h. Cells were subsequently harvested and total RNA extracted. Levels of HIV-1 US, SS and MS RNAs was determined by qRTPCR as outlined. Shown is the result of N = 3 independent assays [file 12977_2017_330_MOESM13_ESM.tif]
